# Updating the evolutionary history of Carnivora (Mammalia): a new species-level supertree complete with divergence time estimates

**DOI:** 10.1186/1741-7007-10-12

**Published:** 2012-02-27

**Authors:** Katrin Nyakatura, Olaf RP Bininda-Emonds

**Affiliations:** 1 Institute for Systematic Zoology and Evolutionary Biology, Friedrich-Schiller-Universität Jena, Erbertstrasse 1, 07743 Jena, Germany; 2 AG Systematics and Evolutionary Biology, IBU-Faculty V, Carl von Ossietzky Universität Oldenburg, Carl von Ossietzky Strasse 9-11, 26111 Oldenburg, Germany

**Keywords:** Carnivora, conservation biology, divergence times, diversification, macroevolution, phylogenetic supertrees, supermatrix, timetree

## Abstract

**Background:**

Although it has proven to be an important foundation for investigations of carnivoran ecology, biology and evolution, the complete species-level supertree for Carnivora of Bininda-Emonds *et al*. is showing its age. Additional, largely molecular sequence data are now available for many species and the advancement of computer technology means that many of the limitations of the original analysis can now be avoided. We therefore sought to provide an updated estimate of the phylogenetic relationships within all extant Carnivora, again using supertree analysis to be able to analyze as much of the global phylogenetic database for the group as possible.

**Results:**

In total, 188 source trees were combined, representing 114 trees from the literature together with 74 newly constructed gene trees derived from nearly 45,000 bp of sequence data from GenBank. The greater availability of sequence data means that the new supertree is almost completely resolved and also better reflects current phylogenetic opinion (for example, supporting a monophyletic Mephitidae, Eupleridae and Prionodontidae; placing *Nandinia binotata *as sister to the remaining Feliformia). Following an initial rapid radiation, diversification rate analyses indicate a downturn in the net speciation rate within the past three million years as well as a possible increase some 18.0 million years ago; numerous diversification rate shifts within the order were also identified.

**Conclusions:**

Together, the two carnivore supertrees remain the only complete phylogenetic estimates for all extant species and the new supertree, like the old one, will form a key tool in helping us to further understand the biology of this charismatic group of carnivores.

## Background

Carnivora (lions, tigers and bears, among others) represent a medium-sized order within Mammalia. It is noteworthy for the charismatic appeal of many of its members as well as the large diversity harbored within it. With its inclusion of both terrestrial and aquatic species, Carnivora is one of few mammalian orders to occur naturally on all the continents. It also presents one of the largest size ranges of any mammalian order among its extant representatives at some five to six orders of magnitude between the Least Weasel (*Mustela nivalis*, 35 to 250 g) and the Southern Elephant Seal (*Mirounga leonina*, 2200 to 5000 kg).

With its publication, the carnivore supertree of Bininda-Emonds *et al*. [1] provided the first complete species-level phylogeny of this diverse mammalian order that was based on a robust, repeatable methodology. In the intervening 10 years, the carnivore supertree has formed the basis for numerous studies illuminating the biology of this group, including its macroevolution and conservation biology (for example, [2-4]); morphological, molecular and behavioral evolution (for example, [5-8]); and disease and parasite risk (for example, [9,10]). Although the method used to construct the tree, supertree construction (*sensu *[11]), was controversial at the time and arguably remains so to this day, the same cannot be said for the phylogenetic relationships presented in the supertree, which largely mirrored the current opinion of the day accurately (for example, compare with [12]). That being said, the pattern of relationships pictured in the carnivore supertree are now out-of-date in several places due to three main factors: taxonomic changes within Carnivora leading to a different set of accepted species; information from additional data sources, primarily DNA sequence data; and methodological limitations in the original analysis.

The original carnivore supertree was based on the 273 species recognized by Wozencraft [13]. In the meantime, however, the number of recognized species has increased to at least 286 [14], in part due to new discoveries, but largely due to changes in taxonomic opinion resulting in both the splitting and lumping together of previous species (but with more of the former).

At the time that data collection for the initial carnivore supertree was concluded (January 1996), the molecular revolution was still in its infancy. The amount of DNA sequence data for the group available in GenBank amounted to only 677 sequences for 48 species [15]. By March 2004, the data set had increased to 1,984,623 sequences for 197 species [15] and by December 2007 it had increased further to cover a total 248 species. (The number of sequences becomes difficult to compare because of the fusion of accessions by the National Center for Biotechnology Information. For instance, > 99% of the nearly two million sequences from 2004 derive from the domestic dog genome project and are now superseded by the genome sequences for that species.) Although molecular data have largely reaffirmed phylogenetic relationships within Carnivora obtained using phenotypic data, they have also toppled some long-held traditional groupings and sets of relationships. Particularly noteworthy changes include Mephitidae being elevated out of Mustelidae [16,17]; *Nandinia *forming the sister group to all remaining feliform carnivores [18]; *Prionodon *being more closely related to Felidae than to Viverridae [19]; and the monophyly of the Malagasy Viverridae and Herpestidae as Eupleridae [20]. Of these now widely accepted hypotheses, robust evidence was only available for the first two in 1996 (for example, [16,21-27]), and only starting to gain acceptance among carnivore systematists.

Finally, the analyses for the carnivore supertree were also hindered by several methodological limitations. First, a number of assumptions of monophyly were made, in part for computational reasons. Because an analysis of 273 species simultaneously was impractical at the time, a compartmentalized approach (*sensu *[28]) was taken instead, such that the supertree was a composite formed from a family-level supertree to which individual supertrees for each of the families (and Lutrinae and Mephitinae within Mustelidae) were grafted. Thus, the monophyly of these groups could not be contradicted, even if some evidence to the contrary existed at the time (for example, as for *Nandinia *with respect to Viverridae). In addition, where a source tree contained a higher-level taxon as a terminal taxon, the tree was coded as if all constituent species of that taxon were present as an unresolved node. Although this assumption of monophyly could still be contradicted, the monophyly of the higher-level taxon was nonetheless artificially up-weighted through this procedure. Both sets of assumptions now represent avoidable 'appeals to authority' (*sensu *[29]). Second, as correctly pointed out by Gatesy *et al*. [29], there was no attempt to correct for potential data duplication between the source trees, meaning that more commonly used data sources were effectively up-weighted. Finally, the paucity of available sequence data meant that the molecular divergence time estimates were derived by mapping relative branch lengths from the source publications on to the topology of the supertree, even when the two topologies conflicted. Although these shortcomings are real, they fortunately appear to have had little negative impact empirically; as mentioned, both the topology of the tree as well as the estimated divergence time estimates were uncontroversial.

The goal of this study is to produce an updated version of the carnivore supertree that accounts for both current taxonomic opinion and additional data sources (primarily DNA sequence data, but also additional phenotypic data) as well as corrects for the shortcomings present in the original analysis by using the best available methodology and analytical methods. As with the original analysis, supertree construction still represents the only robust methodology able to include as much of the phylogenetic database as possible so as to provide a complete phylogenetic estimate for all extant Carnivora species. We are confident that the updated carnivore supertree will form an important foundation for understanding the biology of this order for some time to come.

## Results and discussion

### Data availability

**Figure 1:**
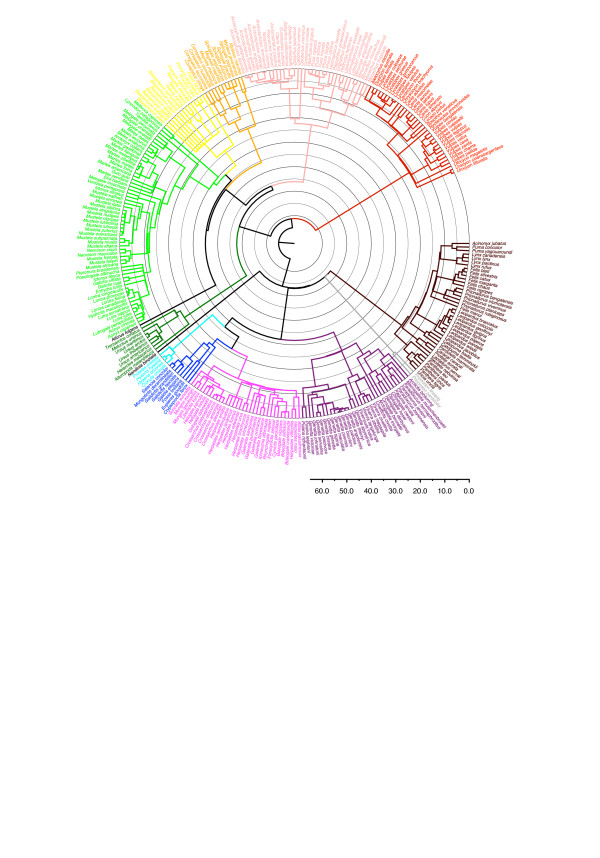
**The complete carnivore supertree showing the best estimates of the divergence times**. The major carnivore lineages counterclockwise from the top left are Canidae (red), Pinnipedia (pink), Mephitidae (orange), Procyonidae (yellow), Mustelidae (light green), Ailuridae (black), Ursidae (dark green), Nandiniidae (black), Hyaenidae (light blue), Eupleridae (dark blue), Herpestidae (light purple), Viverridae (dark purple), Prionodontidae (grey) and Felidae (brown); outgroup taxa have been omitted (see Figure 2). Time intervals are in units of 5 Ma.

The supertree (Figure 1) contains all 286 carnivore species listed in Wozencraft [14]. DNA sequence data from GenBank were only available for 229 of these species (see Additional file 1). For a handful of species (*Bassaricyon lasius, Bdeogale jacksoni, Crossarchus ansorgei, Crossarchus platycephalus, Dologale dybowskii, Galidictis grandidieri, Leopardus braccatus, Lutra nippon, Martes gwatkinsii, Meles leucurus, Melogale everetti, Melogale orientalis, Mustela subpalmata, Neovison macrodon, Poiana leightoni *and *Spilogale angustifrons*), information about their phylogenetic placement was limited to that present in Wozencraft [14]. Often these species were newly recognized compared to Wozencraft [13] and the usable phylogenetic information was limited to their generic membership. Where additional information was found in Wozencraft [14] for these species (for example, *Mustela subpalmata *being recently separated from *Mustela nivalis*, thereby implying a close relationship), this information was included to prevent the poorly known species from collapsing resolution within the genus completely.

Although individual species were present more often in source trees obtained from the literature as opposed to from GenBank (paired sign test *P *= 0.0089), this result derived more from the unequal numbers of trees in each category (244 versus 74, respectively). Correcting the values for this imbalance results in individual species being overrepresented in the GenBank-derived trees (paired sign test *P *< 0.0001). Even so, the histograms for both data sources are similar and right-skewed, indicating that most species are present in only a limited number of source trees, with only a few species being broadly sampled (for example, *Acinonyx jubatus*, which appears in 122 trees and is the best sampled species); the same general pattern is true when both data sources are combined. The GenBank-derived trees are also larger than those from the literature (an average of 53.7 versus 16.7 species, respectively). Indeed the average size for the molecular trees is larger than the largest literature tree (50 species) and the smallest molecular tree (*CYP1A1*; 22 species) is larger than the average size for the literature trees. The increasing ease with which sequence data can be obtained means that this trend will likely become even more strongly expressed in the future.

### General structure of the supertree

Summary statistics for the carnivore supertree (node ID number, node age and nodal support) can be found in Additional file 2. Overall, the tree is much better resolved than the original supertree, with only nine polytomies in total for a greater total number of species (92.6% versus 78.1% resolved, respectively). In addition, the overall topology of the supertree demonstrated good support with respect to the set of 315 source trees (literature plus gene trees combined). Across the entire tree, the average reduced qualitative support index (rQS) was 0.233 (± a standard error of 0.174), indicating that most nodes were supported by more source trees than they were contradicted by. There were no novel clades, either in the sense that a given clade was contradicted by all source trees (*sensu *[30]) or not supported by any source trees (*sensu *[31]). Finally, 53 of the 264 ingroup nodes were not contradicted by any source trees and were supported by an rQS of 1.0. These latter clades tended to be small, containing 3.1 species on average; however, Herpestidae with its 33 species also fell into this category.

**Figure 2:**
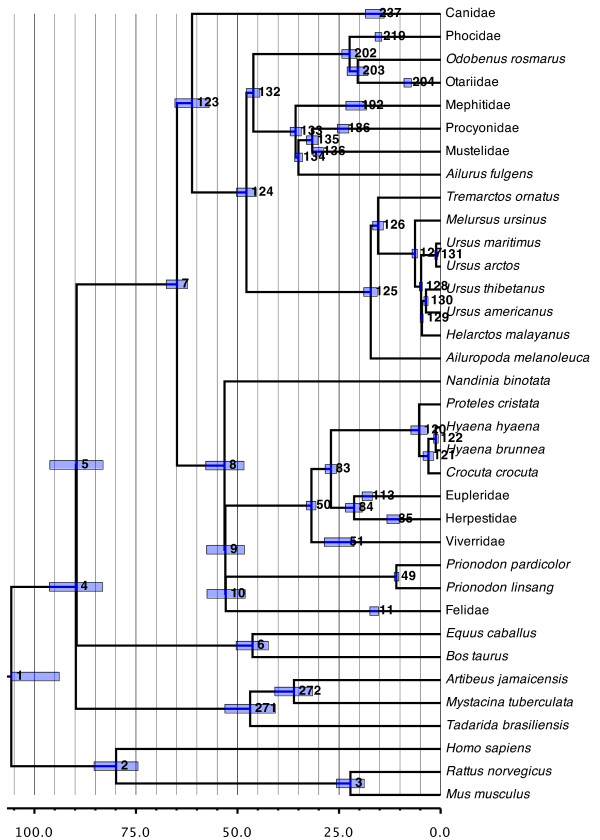
**Phylogenetic relationships and divergence-time estimates among the major carnivore lineages and within the smaller families**. Nodes are individually numbered, with node bars indicating 95% confidence intervals on the divergence time estimates (in Ma before present).

At the family level (Figure 2), the current supertree presents a number of relationships that conflict with those from the supertree of Bininda-Emonds *et al*. [1] but are identical with those in the recent family-level supermatrix study of Meredith *et al*. [32]. These include the polyphyly of the traditional Mustelidae into Mephitidae and Mustelidae *sensu stricto*; the polyphyly of traditional Viverridae into Eupleridae (in part), *Nandinia, Prionodon *and Viverridae *sensu stricto*; and the paraphyly of traditional Herpestidae with inclusion of former viverrid-assigned members of Eupleridae (*Cryptoprocta *and *Fossa*). In all cases, these relationships were previously prohibited through the assumptions of (family) monophyly made in the original analysis. However, they accurately reflect current opinion regarding a separate family-level status for Eupleridae, Mephitidae, Nandiniidae [14] and possibly Prionodontidae [19] (for an overview of higher-level relationships in general, see [18]). These changes notwithstanding, higher-level relationships within Caniformia are otherwise identical to those presented in the original carnivore supertree. The positioning of Mephitidae as the sister group to the remaining Musteloidea, with the Red Panda, *Ailurus fulgens*, being the sister group to the resulting clade matches that obtained by Flynn *et al*. [18]. The relationships within Feliformia have changed to now place Felidae as the sister group to (Viverridae, (Herpestidae, Hyaenidae)) instead of to Hyaenidae alone as in the original version. However, the relationships among, if not the identities and composition of, the major feliform taxa have historically been contentious and difficult.

The base of the carnivore radiation occurred shortly after the Cretaceous-Paleogene (K-Pg) boundary 64.9 million years (Ma) ago (95% confidence interval (CI), 62.3 to 67.5 Ma). These dates would indicate that the upper constraint of 63 Ma ago used by Springer *et al*. [33] (see Table 1) for the root of crown-group Carnivora might be slightly too young, although the fit between the molecular and fossil data in this case is quite good. Our inferred value for the root also corresponds closely with that of Bininda-Emonds *et al*. [34] (67.1 ± 3.8 Ma) and is only slightly older than those inferred by either Eizirik *et al*. [35] (51.6 to 64.7 Ma) or Springer *et al*. [33] (55.1 ± 4.8 Ma). In total, a post-K-Pg radiation of crown-group Carnivora seems reasonably certain. The origins of the lineage leading to Carnivora are older, at most 89.6 Ma (83.1 to 96.2 Ma) and probably younger given that our analyses did not include the putative sister group of Carnivora (Pholidota; see [36]). The general compression of the divergence times in this area of the supertree lends more evidence to an apparent adaptive radiation near the base of the Laurasiatheria, with the dates inferred here reflecting those indicated by Bininda-Emonds *et al*. [34,37].

**Table 1:** Calibration data used to estimate divergence times within the carnivore supertree.

Clade or split	Age of fossil or range of split (in Ma)	Fossil or split	Reference
Boreoeutheria (1)	96.1 ± 10%	Laurasiatheria versus Euarchontoglires	[37]
Rodentia (2)	57.25	*Tribosphenomys minutus*	[37]
*Rattus *versus *Mus *(3)	12.0	Split between *Rattus *and *Mus*	[33]
Chiroptera (4)	52.2	*Ageina tobieni*	[37]
Cetartiodactyla versus Perissodactyla (5)	52.2	*Diacodexis *sp., *Orientolophis *sp., *Pakicetus inachus*	[37]
Carnivora (7)	50.0	Split between Caniformia and Feliformia	Modified from [33]
Felidae (10)	31.15	*Proailurus jordan*i	[37]
Viverridae (50)	25.72	*Palaeoprionodon lamandini*	[37]
Hyaenidae (83)	19.5	*Herpestides *sp.	OBE (unpublished)
Canidae (123)	43.35	*Procynodictis vulpiceps*	[37]
Ursidae (124)	19.5	*Ursavus brevirhinus*	[37]
Phocidae (202)	19.5	*Pinnarctidion bishopi*	[37]

Unless otherwise indicated, point estimates were also taken to be minimum age constraints. For date ranges, the initial calibration point was the midpoint of the range.

Within Carnivora, most families have relatively deep roots, especially in relation to the basal split within the crown groups (see Table 2). The oldest crown family is Mustelidae (30.1 Ma ago; 95% CI, 28.9 to 31.4 Ma ago), with the crown groups of eight of the 12 remaining families initially radiating between 15 to 25 Ma ago. The only exceptions to the latter are Herpestidae (11.6 Ma ago; 95% CI, 10.2 to 13.2 Ma ago), Otariidae (8.1 Ma ago; 95% CI, 7.2 to 9.0 Ma ago), and the small families Hyaenidae (5.3 Ma ago; 95% CI, 3.3 to 7.3 Ma ago) and Prionodontidae (10.9 Ma ago; 95% CI, 10.3 to 11.4 Ma ago). Although there is a tendency for older family crown groups to also possess more extant species (r = 0.544; *P *= 0.0546), no significant associations existed between the size of the family crown group and either the time of origin of the lineage (r = 0.104; *P *= 0.7338) or the relative amount of lag time between the origin of the lineage and the radiation of the crown group (r = -0.370; *P *= 0.2134). The dates inferred in this study for the carnivoran families are generally significantly older than those in Eizirik *et al*. [35], although estimates for the basal radiations of Eupleridae, Mephitidae and Phocidae agree strongly between the studies.

**Table 2:** Estimated divergence times relating to the major carnivore lineages.

Taxon	Node ID	Divergence time in Ma with 95% CI	Time of origin in Ma with 95% CI	% lag	Number of species	Total PD	Average PD per species
Carnivora	7	64.9 (62.3 to 67.5)	89.6 (83.1 to 96.2)	27.6	286	2,426.0	8.5
Feliformia	8	53.2 (48.4 to 57.8)	64.9 (62.3 to 67.5)	18.0	121	1,047.4	8.7
Nandiniidae			53.2 (48.4 to 57.8)		1	53.2	53.2
Felidae	11	16.3 (15.3 to 17.4)	52.9 (48.1 to 57.5)	69.2	40	282.1	7.1
Prionodontidae	49	10.9 (10.3 to 11.4)	52.9 (48.1 to 57.5)	79.4	2	63.8	31.9
Viverridae	51	23.6 (21.3 to 28.6)	31.8 (30.7 to 33.0)	25.8	33	277.4	8.4
Herpestidae	85	11.6 (10.2 to 13.2)	21.3 (19.3 to 23.4)	45.5	33	208.2	6.3
Eupleridae	113	18.0 (16.8 to 19.3)	21.3 (19.3 to 23.4)	15.5	8	82.5	10.3
Hyaenidae	120	5.3 (3.3 to 7.3)	27.0 (25.6 to 28.4)	80.4	4	36.5	9.1
Caniformia	123	61.2 (57.0 to 65.4)	64.9 (62.3 to 67.5)	5.7	165	1,353.9	8.2
Ursidae	125	17.2 (15.6 to 18.9)	47.8 (45.4 to 50.2)	64.0	8	100.8	12.6
Ailuridae			35.0 (34.0 to 35.9)		1	35.0	35.0
Mustelidae	136	30.1 (28.9 to 31.4)	31.6 (30.2 to 33.0)	4.7	59	368.8	6.3
Procyonidae	186	24.0 (22.6 to 25.4)	31.6 (30.2 to 33.0)	24.1	14	194.2	13.9
Mephitidae	192	20.7 (18.2 to 23.3)	35.7 (34.3 to 37.0)	42.0	12	123.3	10.3
Odobenidae			20.4 (17.9 to 23.0)		1	20.4	20.4
Otariidae	204	8.1 (7.2 to 9.0)	20.4 (17.9 to 23.0)	60.3	16	65.7	4.1
Phocidae	219	15.3 (14.5 to 16.1)	22.4 (20.6 to 24.3)	31.7	19	126.8	6.7
Canidae	237	16.3 (13.9 to 18.5)	61.2 (57.0 to 65.4)	73.4	35	259.9	7.4

Complete information for all nodes in the tree can be found in Additional file 2.

CI: confidence interval; PD: phylogenetic diversity

For Canidae and Felidae, the long lag times between the origins of both families and their basal radiations reflect the pattern of competitive subclade replacement, with each of the crown groups being the latest in a series of radiations along each lineage (see [38,39]). By contrast, the small and recent radiation for Hyaenidae disguises that this family was much more successful in the past (see [40]); the same holds true for the monotypic Odobenidae (see [41]). Of the remaining monotypic families, Ailuridae and especially Nandiniidae represent the last relics of ancient lineages that originated 35.0 Ma ago (95% CI, 34.0 to 35.9 Ma ago) and 53.2 Ma ago (95% CI, 48.4 to 57.8 Ma ago), respectively; the age for Ailuridae is slightly older than that inferred in the original supertree (29.3 Ma).

### Relationships and divergence times within the higher carnivore taxa

#### Canidae (Figure 3)

**Figure 3:**
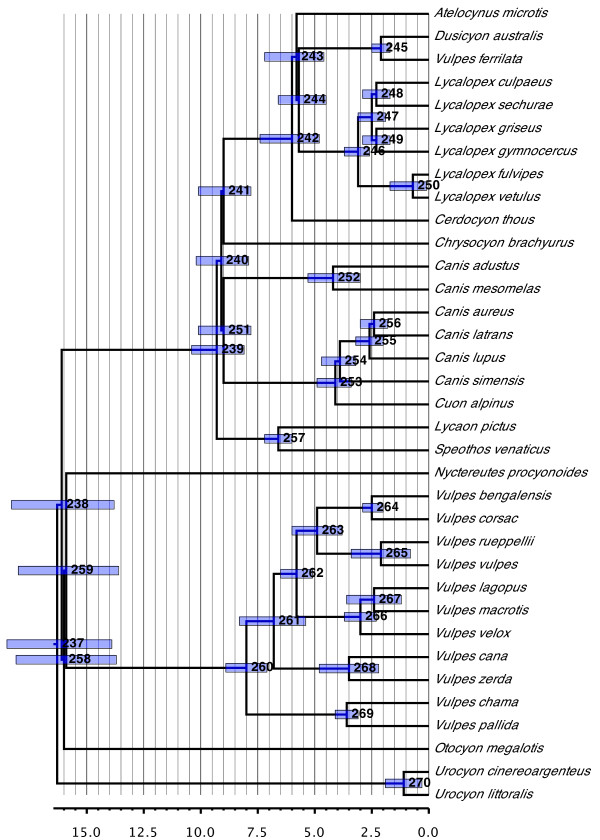
**Phylogenetic relationships and divergence times estimates within Canidae**. Nodes are individually numbered, with node bars indicating 95% confidence intervals on the divergence time estimates (in Ma before present).

The family is broadly divided into dog-like and fox-like clades (Canini and Vulpini, respectively [42]). Of the three genera that have been difficult to place historically, *Nyctereutes, Otocyon *and *Urocyon*, the former two form successive sister taxa to the remaining Vulpini, whereas the latter forms the sister group to all remaining canids (in agreement with [43-47]). However, all the genera represent long-branch taxa (range = 15.9 to 16.3 Ma old) that together with Canini and Vulpini diverge at the base of the canids in an exceedingly short time span (< 0.5 Ma). These facts help explain the historical difficulty in determining the phylogenetic affinities of these taxa as well as throw caution on the placements inferred herein. Indeed, except for the placement of *Urocyon*, all relevant nodes in this region of the tree are characterized by highly negative rQS values, indicating substantial disagreement among the source trees. The placement of these taxa, however, does agree with those recovered by Lindblad-Toh *et al*. [45], among others.

Within each of Canini and Vulpini, the recovered relationships agree strongly with those recovered by a variety of other authors. This includes the paraphyly of *Canis *due to the inclusion of *Cuon alpinus *(also [44,45]) and the polyphyly of the jackals, with *Canis aureus *being more closely related to *Canis latrans *than to the remaining jackals (*Canis adustus *and *Canis mesomelas*) (also [44]). Unusual here, however, is that *Canis latrans *and *Canis lupus *do not form sister taxa as is commonly recovered. Otherwise, the only other major differences within Canini compared to Lindblad-Toh *et al*. [45] and Zrzavy and Ricankova [44] are that *Lycaon pictus *and *Speothos venaticus *form a clade that is the sister group to all remaining Canini instead of clustering within *Canis *(*sensu lato*) and the South American foxes (*sensu lato*), respectively, the latter typically being sister to *Chrysocyon brachyurus*. The relationships we recovered within Vulpini are identical to those found by Lindblad-Toh *et al*. [45] after accounting for differences in species sampling. An unexpected result, however, is that *Vulpes velox *and *Vulpes macrotis *are not sister species, given the long-standing question as to whether or not they are indeed separate species (for example, compare the two taxonomies [13] and [14] from Wozencraft). Instead, the latter forms a clade with *Vulpes lagopus*.

One obvious inaccuracy in the tree is the clustering of *Vulpes ferrilata *with *Dusicyon australis *within the South American fox clade, a placement that derives from the paucity of phylogenetic information for the former species. At the time of our analyses, no usable sequence data for *Vulpes ferrilata *were present in GenBank (the existing *MT-CYB *sequence being too short and non-overlapping) and the phylogenetic relationships of the species were specified in only one tree in the literature, that of Clutton-Brock *et al*. [48]. This latter study represents a phenetic analysis of 92 morphological characters that postulates what many would view as non-conventional relationships within Canidae. However, it represents the only more-or-less rigorous study to include this species (the remaining studies being taxonomies, which were discarded) and the placement of *Vulpes ferrilata *in the supertree accurately reflects its placement in this study.

#### Pinnipedia (Figure 4)

**Figure 4:**
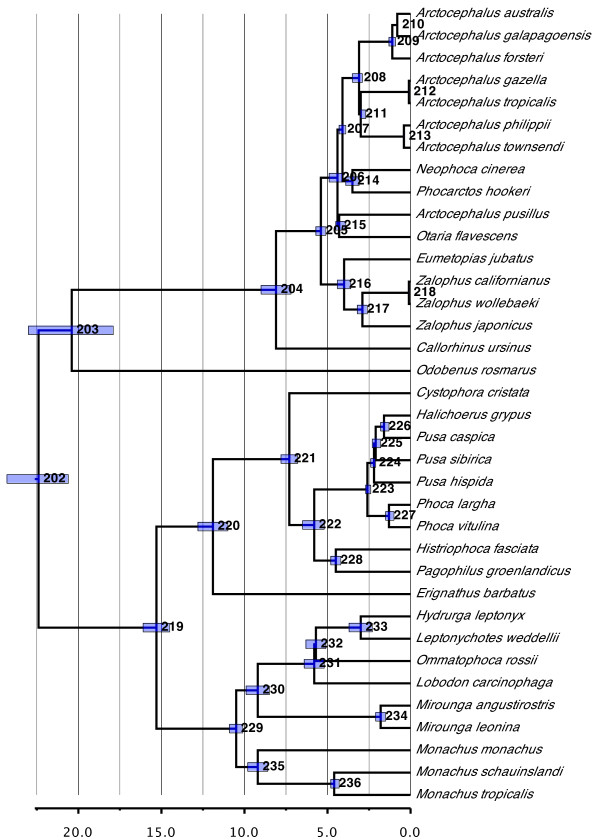
**Phylogenetic relationships and divergence times estimates within Pinnipedia (Odobenidae, Otariidae, and Phocidae)**. Nodes are individually numbered, with node bars indicating 95% confidence intervals on the divergence time estimates (in Ma before present).

Phylogenetic relationships within Phocidae reflect the more-or-less long-standing consensus picture for this group (see [49]), with the true seals being divided into the northern Phocinae and southern Monachinae. The only substantial difference with the original supertree involves slight alterations within Lobodontini (*Hydrurga, Leptonychotes, Lobodon*, and *Ommatophoca*). However, nearly all variations within this tribe have been proposed at some point (for example, compare [50-52]), indicating a lack of resolution. *Halichoerus grypus *remains problematic, at least taxonomically. Our inferred placement of *Halichoerus *as the sister species of *Pusa caspica *agrees with several recent studies ([50,52,53]; but see [51]) and reflects historical arguments that *Halichoerus *clusters within *Phoca *(*sensu lato; Histriophoca, Pagophilus, Phoca *and *Pusa*) [54-56]. Yet, in spite of the long-standing, overwhelming evidence and calls to the contrary, *Halichoerus *has somehow always managed to retain its distinct generic status. The resolution to this problem depends to some degree on what should be done with *Phoca *(*sensu lato*), which varies regularly between being recognized as a single genus (for example, [13,57]), as four separate genera (for example, [14,53,58]) or some intermediate solution (for example, [54]). Given the relative divergence times inferred in this and other studies (for example, [49,50,59]), we would argue that *Histriophoca *and *Pagophilus *should remain as distinct genera, with both *Halichoerus *and *Pusa *being subsumed with *Phoca*. In the latter case, the designation of subgenera within *Phoca *is difficult due to the paraphyly of *Pusa*, unless one is willing to subsume *Halichoerus *within the subgenus *Pusa*.

*Odobenus *is placed as the sister taxon to Otariidae, in agreement with the majority of morphological and molecular evidence ([50-52,60]; but see [61]). As mentioned, Otariidae represent a recent radiation and display a distinct lag time between their origin and initial diversification (20.4 versus 8.1 Ma ago, respectively). *Callorhinus *occupies its traditional position as the sister group of all remaining species. Thus, a clear distinction between sea lions (Otariinae) and fur seals (Arctocephalinae) remains missing, reflecting the weak evidence upon which it was originally proposed (presence versus absence of abundant underfur; see [62]). Moreover, Otariinae and the remaining Arctocephalinae themselves are also paraphyletic, with several genera (*Neophoca, Otaria *and *Phocarctos*) being more closely related to *Arctocephalus *than to the remaining sea lion genera (*Eumetopias *and *Zalophus*). Reflecting numerous molecular studies [50,52,63,64], *Arctocephalus *is not monophyletic, with *Arctocephalus pusillus *and *Otaria flavescens *forming a clade and the clade of *Neophoca *+ *Phocarctos *forming the sister group to the remaining species of *Arctocephalus*. All species are well sampled, which, together with similar results in other studies, reduces the likelihood that the result is artifactual. However, because no clear consensus yet exists as to relationships among *Arctocephalus *and Otariinae (compare the above studies), taxonomic changes for the *Arctocephalus *species (for example, resurrection of *Arctophoca *for some species; [65]) seem premature. Much of the uncertainty here might derive from the group (*Arctocephalus *+ *Neophoca *+ *Otaria *+ *Phocarctos*) representing a very recent radiation that is not more than 4.4 Ma old (95% CI, 4.1 to 4.97 Ma). Several very recent speciation events are also inferred within the group (for example, between *Arctocephalus gazella *and *Arctocephalus tropicalis *or between *Arctocephalus philippii *and *Arctocephalus townsendi*) as well as between *Zalophus californianus *and *Zalophus wollebaeki*.

#### Mephitidae (Figure 5)

**Figure 5:**
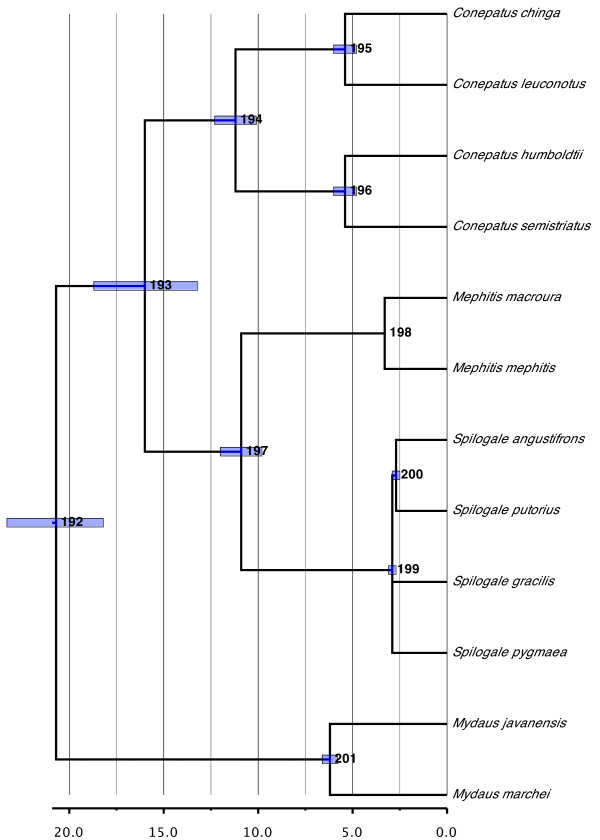
**Phylogenetic relationships and divergence times estimates within Mephitidae**. Nodes are individually numbered, with node bars indicating 95% confidence intervals on the divergence time estimates (in Ma before present).

The taxonomic status of Mephitidae as a separate family is supported, with the clade containing the Old World stink badgers (genus *Mydaus*) as the sister group to the remaining New World skunks (genera *Conepatus, Mephitis*, and *Spilogale*). The split between these two groups is inferred at 20.7 Ma ago (95% CI, 18.2 to 23.3 Ma ago). Within the New World skunks, the monophyly of each genus is supported and phylogenetic relationships also roughly follow biogeographical patterns, with the more largely South American genus *Conepatus *forming the sister group to the clade of *Mephitis *+ *Spilogale*, with its more Central and North American distribution. The age of the split between these two groups (16.0 Ma ago; 95% CI, 13.2 to 18.7 Ma ago) as well as of the radiation of *Conepatus *(11.2 Ma ago; 95% CI, 10.1 to 12.3 Ma ago) would indicate a re-invasion of South America by the lineage leading to *Conepatus *well before the establishment of the Panamanian land bridge about 3 Ma ago.

#### Procyonidae (Figure 6)

**Figure 6:**
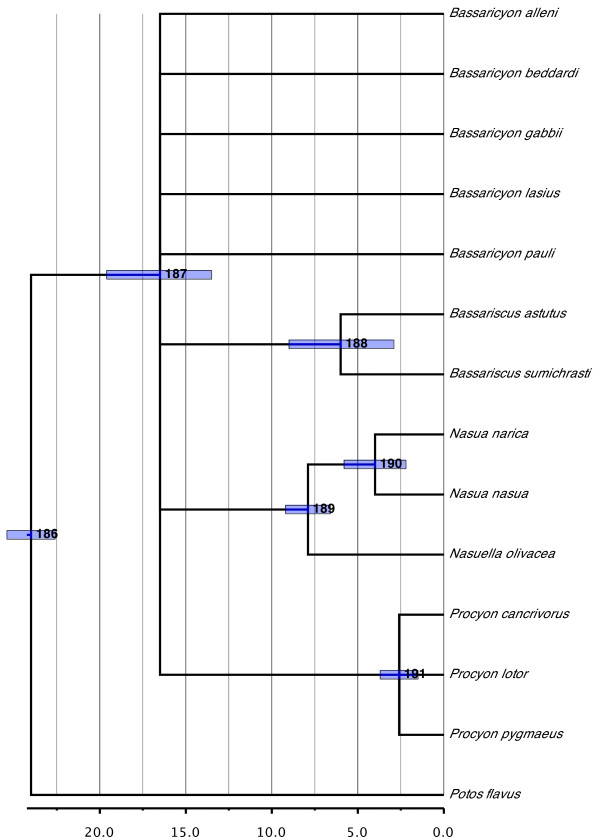
**Phylogenetic relationships and divergence times estimates within Procyonidae**. Nodes are individually numbered, with node bars indicating 95% confidence intervals on the divergence time estimates (in Ma before present).

Procyonidae, and the genus *Procyon *in particular, have undergone some of the largest alpha taxonomic changes since the publication of the original supertree, with four previously recognized species of *Procyon *(*gloveralleni, insularis, maynardi *and *minor*) being subsumed into *Procyon lotor*. Relationships among the remaining three *Procyon *species, however, remain unresolved and represent a very recent radiation (2.6 Ma ago; 95% CI, 1.5 to 3.7 Ma ago).

Resolution within Procyonidae is poor and worse than in the original version of the supertree, where only the intrageneric relationships were unresolved. Apart from *Potos flavus *being inferred as the sister group to the remaining species and a sister-group relationship between *Nasua *and *Nasuella*, little structure is to be found. Much of the lack of resolution probably derives from *Bassaricyon*, which was not reconstructed as monophyletic. Only two species show reasonable sampling coverage for this genus, *Bassaricyon alleni *and *Bassaricyon gabbii*. The remaining three species (and *Bassaricyon lasius *in particular, which was only included in [14]) are therefore less constrained in their positioning, leading to possible losses of resolution.

#### Mustelidae (Figure 7)

**Figure 7:**
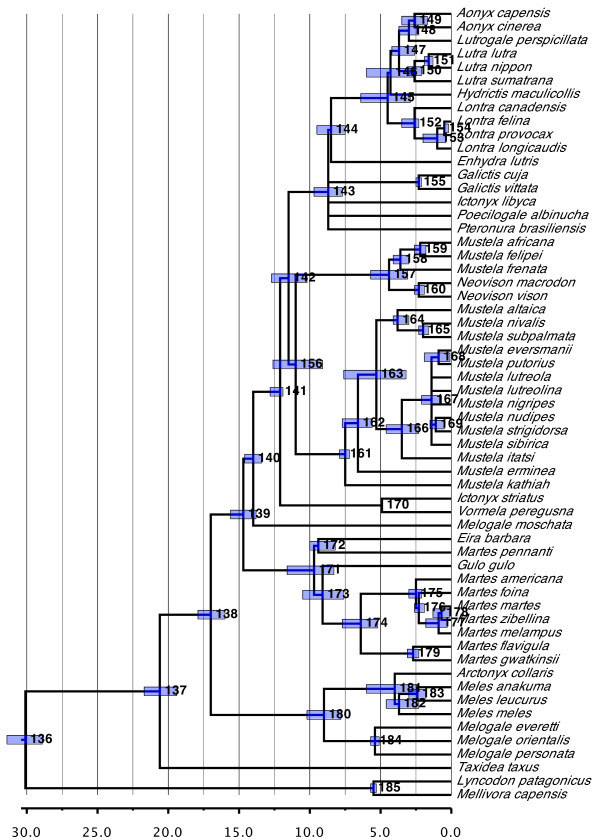
**Phylogenetic relationships and divergence times estimates within Mustelidae**. Nodes are individually numbered, with node bars indicating 95% confidence intervals on the divergence time estimates (in Ma before present).

The subtree for Mustelidae displays the greatest concentration of non-monophyletic taxa across the carnivores. Some of these instances represent areas of continuing dispute (for example, *Neovison *with respect to *Mustela*) or growing consensus pointing to the need for future revision (for example, *Martes *with respect to *Martes pennanti*). Badgers ('Melinae') in particular are confirmed as polyphyletic. As in the original supertree, most species of badger appear close to the base of the family. Together with the separation of *Mydaus *into Mephitidae, this positioning hints that the diagnostic badger characters may represent plesiomorphic traits for musteloid-like carnivores.

Other instances of non-monophyly may derive from limitations in the underlying data set. Thus, *Melogale *is probably polyphyletic because the two main species with the greatest data coverage, *Melogale moschata *and *Melogale personata*, only co-occur on the heavily down-weighted seed tree. The same is true of the two species of *Ictonyx*, causing in part the polyphyly of Galictinae. Lutrinae may be non-monophyletic, with 62% of the equally most parsimonious trees from the matrix representation parsimony (MRP) analysis speaking against the monophyly of the group. Interestingly, the sequence data are responsible for this result. Most relevant literature trees place *Pteronura brasiliensis *either within otters or as part of a polytomy with other otter species. By contrast, four of the six individual genes trees (*GHR, MT-CYB, MT-TP *(tRNA-Pro), and *MT-TT *(tRNA-Thr)) cluster *Pteronura brasiliensis *with non-otters, a result that is also reflected in the supermatrix analysis of the sequence data (see below). Of these four genes, only the *MT-CYB *accession is potentially problematic, with all the top BLAST hits being non-otters. It is difficult to envisage a case of taxonomic misidentification in this instance, especially given the source of the data [66], although an accidental mix up among the samples cannot be ruled out. This same sequence, however, was also used in a later analysis by the same authors [67], with no ill effects.

Otherwise, the relationships reconstructed for Mustelidae generally agree with those found by Koepfli *et al*. [67] apart from the indicated polyphyly of Galictinae. Our analyses, however, generally reconstructed older divergence times across the group than those inferred by Koepfli *et al*. [67].

#### Ursidae (Figure 2)

As in the original supertree, *Ailuropoda melanoleuca *forms the sister group to the remaining species of Ursidae, with the long branch upon which it sits being somewhat shorter than inferred previously (17.2 Ma; 95% CI, 15.6 to 18.9 Ma versus 21.8 Ma). *Tremarctos ornatus *is firmly placed as the sister group to a monophyletic Ursinae. Relationships within the latter remain problematic and our inferred topology, which does not support the monophyly of *Ursus*, reflects merely another in a long line of conflicting hypotheses (compare [47,64,68,69]). The difficulties here undoubtedly stem from an inferred adaptive radiation for the group some 5 Ma ago [68], a result and value close to that recovered here (6.3 Ma ago; 95% CI, 5.7 to 7.0 Ma ago). Because even the application of ever increasing amounts of molecular sequence data do not seem to be providing a consensus topology for Ursinae, an alternative approach involving meta-genomic data (for example, rare genomic changes such as SINEs or chromosomal rearrangements) might be needed instead to bring resolution to this problem.

#### Hyaenidae (Figure 2)

Hyaenidae display one of the longer lag times between their origin and the diversification of the crown group (27.0 versus 5.3 Ma, respectively), reflecting that the modern species represent the relict of a much larger, historical radiation. Both of these date estimates are up to 5 Ma younger than those found by Koepfli *et al*. [70] or Eizirik *et al*. [36]. The inferred relationships reflect current opinion for the group, with *Proteles cristata *and *Crocuta crocuta *forming successive sister species to the remaining species [70], which alternate between being placed in the same (*Hyaena*; [13]) or different genera (*Hyaena *and *Parahyaena*; [14]).

#### Eupleridae (Figure 8)

**Figure 8:**
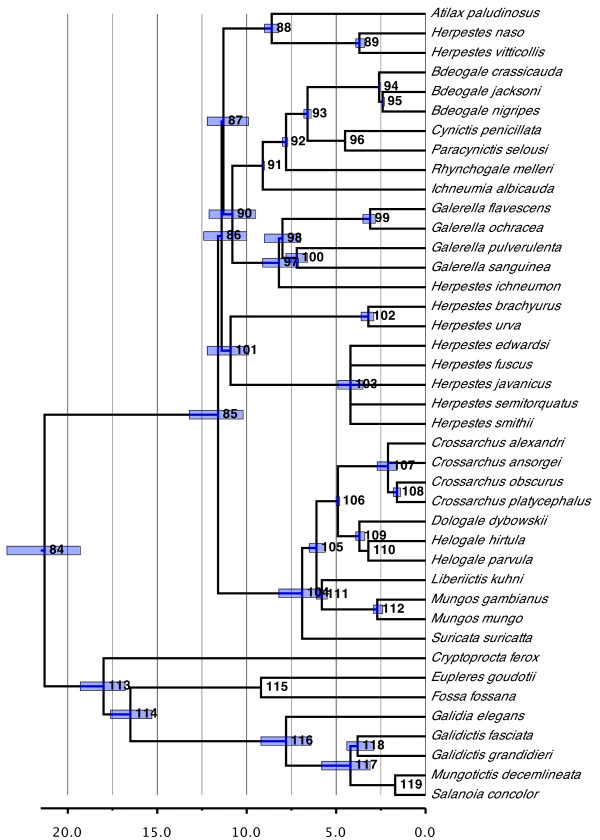
**Phylogenetic relationships and divergence times estimates within Eupleridae and Herpestidae**. Nodes are individually numbered, with node bars indicating 95% confidence intervals on the divergence time estimates (in Ma before present).

The dispersal of Eupleridae to Madagascar is inferred to have occurred sometime between 21.3 and 18.0 Ma ago (the times for the origin of and basal radiation within the family, respectively). These dates agree with the estimates of Yoder *et al*. [20] and Gaubert and Veron [19] and therefore support their hypothesis of a single invasion from the African continent as the most likely biogeographic hypothesis for this family (also [71]).

Our analyses reconstruct those euplerid genera formerly associated with Herpestidae (Galidinae: *Galidia, Galidictis, Mungotictis *and *Salanoia*) as a monophyletic group about 7.8 Ma old (95% CI, 6.4 to 9.2 Ma old); the recovered relationships generally agree with those of Yoder *et al*. [20]. By contrast, the remaining genera that were associated previously with Viverridae ('Euplerinae': *Cryptoprocta, Eupleres *and *Fossa*) are not monophyletic. This result also agrees with the few others that include all these species [20,47], although the inter-relationships differ among the studies and therefore remain poorly resolved (also [71]).

The apparent monophyly of Eupleridae would necessitate the convergent derivation of a herpestid-like auditory bulla in Galidinae, the viverrid-like bulla of 'Eupleridae' being most parsimoniously reconstructed as a symplesiomorphy. This is unexpected given that the morphology of the auditory bulla is commonly held to be highly conserved and taxon-specific among carnivores. However, other extreme instances of morphological convergence are well documented among feliform carnivores in particular (see [19,72]).

#### Herpestidae (Figure 8)

Relationships within Herpestidae are much better resolved compared to the original supertree, the only remaining areas of poor resolution involving five species of *Herpestes *and two of *Crossarchus*. This improved state of affairs derives from the availability, even if meager, of molecular sequence data for *Liberiictis kuhni, Rhynchogale melleri *and several species of *Herpestes*, species for which virtually no phylogenetic information was available in 1996. *Dologale dybowskii*, however, remains problematic, with no recent available data other than the statement in Wozencraft [14] that it is believed by most to be the sister group to *Helogale*.

A clear split between the social (*Crossarchus, Dologale, Helogale, Liberiictis, Mungos *and *Suricata*) and solitary mongooses (*Atilax, Bdeogale, Cynictis, Galerella, Herpestes, Ichneumia, Paracynictis *and *Rhynchogale*) as found by Veron *et al*. [73] and Patou *et al*. [74] is recovered. We reconstructed the clade of solitary mongooses, however, as significantly younger than do Patou *et al*. [74] (11.4 versus 18.5 Ma ago) and also as having undergone a large adaptive radiation at the base of the clade.

With one exception (*Herpestes*), all genera within the family were recovered as monophyletic. That *Herpestes *is split among three independent clades and is therefore polyphyletic is perhaps not surprising (*contra *[73]), given that species of numerous other genera have been assigned to it, even if only in the distant past (for example, *Atilax, Galerella, Helogale *and *Mungos*; see [14]). However, should this general result, which was also found by Patou *et al*. [74] and Agnarsson *et al*. [47], be confirmed, it would necessitate large-scale taxonomic changes for the genus. In particular, *Herpestes *could go from being one of the most species-rich genera within carnivores to a monotypic one given that the type species for the genus (*Herpestes ichneumon*) alone forms the sister taxon of *Galerella *such that, following the suggestion of Patou *et al*. [74], new genus names would need to be found or resurrected for the remaining species.

#### Viverridae (Figure 9)

**Figure 9:**
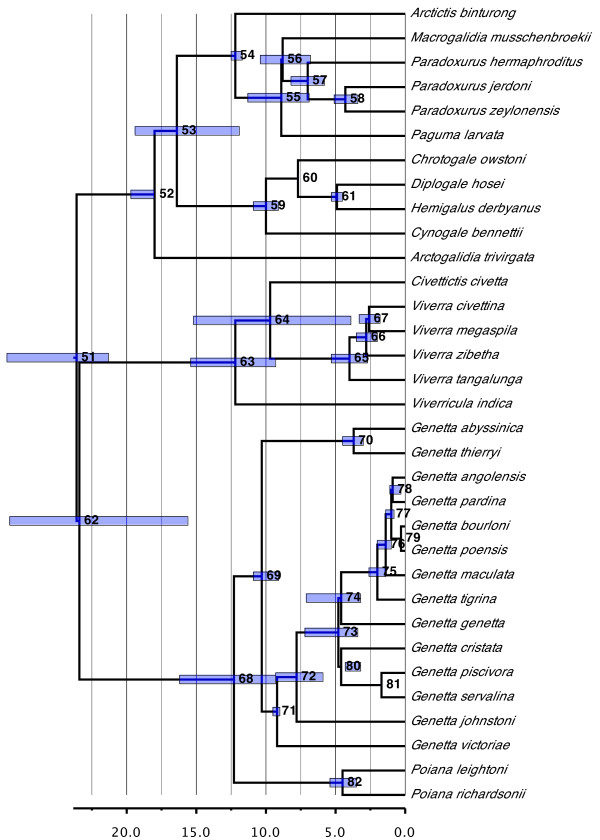
**Phylogenetic relationships and divergence times estimates within Viverridae**. Nodes are individually numbered, with node bars indicating 95% confidence intervals on the divergence time estimates (in Ma before present).

Viverridae are among the oldest of the carnivore families, radiating about 23.6 Ma ago (95% CI, 21.3 to 28.6 Ma ago). The CI includes the value found in the original supertree (27.5 Ma) despite the large taxonomic differences in Viverridae between the two studies (that is, the removal of *Nandinia binotata*, the two *Prionodon *species and the euplerine genera *Cryptoprocta, Eupleres *and *Fossa*). The date also agrees with those obtained by Koepfli *et al*. [70] (25.2 Ma) and Patou *et al*. [75] (26.9 Ma), both of which are much younger than the best estimate of either Gaubert and Cordeiro-Estrela [76] (34.29 Ma) or Eizirik *et al*. [36] (28.6 Ma).

Viverridae split rapidly (within 0.2 Ma) into three major clades, in which the crown groups are well separated from one another temporally. The first (18.0 Ma old; 95% CI, 18.0 to 19.7 Ma) corresponds to the traditional subfamilies Paradoxurinae and Hemigalinae (with all species being found in India and/or Southeast Asia), with Paradoxurinae being rendered as paraphyletic (*contra *[75]). The second and third clades, which are sister to one another, together comprise the traditional Viverrinae, but now correspond, respectively, to Viverrinae (12.2 Ma old; 95% CI, 9.3 to 15.4 Ma old) and Genettinae (12.3 Ma old; 95% CI, 9.3 to 16.2 Ma old) as defined by Gaubert and Cordeiro-Estrela [76]. All the latter species are African in distribution except for the genera *Viverra *and *Viverricula*, which again are Indian and/or Southeast Asian. As postulated by Gaubert and Cordeiro-Estrela [76], an Asian origin for extant viverrids is the most parsimonious solution, with *Civettictis civetta *and Genettinae independently invading Africa.

#### Felidae (Figure 10)

**Figure 10:**
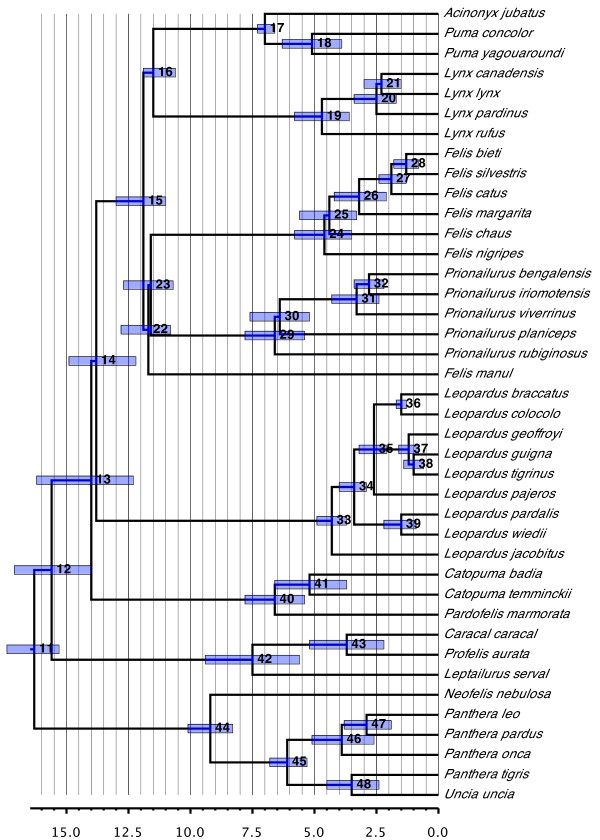
**Phylogenetic relationships and divergence times estimates within Felidae**. Nodes are individually numbered, with node bars indicating 95% confidence intervals on the divergence time estimates (in Ma before present).

Compared to the original supertree, relationships within the Felidae have been turned inside out, with the large cats (the Panthera lineage of [77]) now appearing as the sister group of all remaining felids rather than being deeply nested within the clade. Numerous other differences with the original supertree exist for the inter-relationships of the major felid lineages recognized by O'Brien *et al*. [77] (as well as the phylogenetic status of some). The topology of the supertree, however, agrees strongly with that of Johnson *et al*. [78] with respect to the major field lineages, including supporting the monophyly of all of them. The only differences between the two studies are the reversed positions of the Bay Cat and Caracal lineages as well as *Felis manul *forming the sister group to the combined Domestic Cat and Leopard Cat lineages in the supertree, instead of being a member of the latter. A key difference, however, lies in the inferred divergence times, with those in this study for the major felid nodes being substantially older than those in Johnson *et al*. [78].

Within the felid lineages, only minor differences with respect to Johnson *et al*. [78] are observed. Many of the differences involve groups of species inferred in both studies to have undergone rapid diversifications (for example, within *Lynx *and *Leopardus *and, to a lesser extent, within *Felis *and *Panthera*). Two important differences, however, are that *Felis silvestris *and *Felis catus *do not form a clade in the supertree and that *Leopardus jacobitus *constitutes the sister species to all remaining members of the Ocelot lineage rather than being nested within it.

### Comparison with supermatrix and other supertree analyses

The topology of the supertree is determined primarily by the molecular data set. Comparison of the supertree topology with the supertree obtained only from the molecular gene trees reveals a difference of only 1.1% (with 229 species in common) as measured by a normalized partition metric [79,80] (Table 3; Additional file 3). This stands in sharp contrast to the value of 43.7% (for 265 species) in comparison to the supertree derived from the literature trees only. At least some of these differences, however, derive from the slightly more poorly resolved nature of the latter tree (85.6% versus 98.7%). It is perhaps not surprising, therefore, that the new supertree is also strongly different from that published in 1999 (47.4%; for 235 species), which, in addition to the methodological constraints listed in the Introduction, is more poorly resolved (78.1%) and literature trees contributed more to the underlying database. Many of the important differences between the two versions of the supertree have been detailed above.

**Table 3:** Topological differences between phylogenies derived from various analyses of the carnivore data set.

	1^a^	2^b^	3^c^	4^d^	5^e^	6^f^	7^g^
**1^a^**	-	286	286	229	265	235	229
**2^b^**	0.086	-	286	229	265	235	229
**3^c^**	0.174	0.176	-	229	265	235	229
**4^d^**	0.011	0.106	0.216	-	224	191	229
**5^e^**	0.437	0.432	0.451	0.505	-	224	224
**6^e^**	0.474	0.466	0.494	0.487	0.358	-	191
**7^f^**	0.229	0.240	0.086	0.227	0.541	0.558	-

Differences were quantified by the normalized partition metric [79,80]. Number of shared taxa and partition metric values appear above and below the diagonal, respectively.

^a ^supertree constructed from literature and gene trees; ^b ^supertree constructed from literature, nuclear DNA genes, and mitochondrial DNA supermatrix tree; ^c ^supertree constructed from literature and molecular supermatrix trees; ^d ^supertree constructed from gene trees only; ^e ^supertree constructed from literature trees only; ^f ^supertree from Bininda-Emonds *et al*. [1]; ^g ^supermatrix tree.

**Figure 11:**
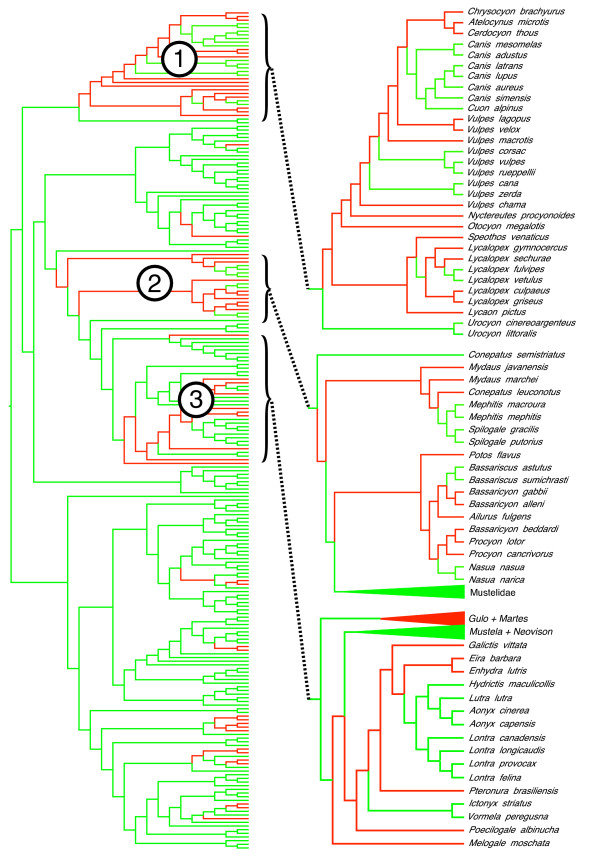
**Phylogenetic relationships among Carnivora as inferred from a partitioned maximum likelihood supermatrix analysis**. The tree includes all 229 carnivore species for which molecular sequence data from the 74 gene data sets were available from GenBank. Taxon names have been omitted for clarity and the orientation of the tree parallels that in Figure 2 as far as possible. Green branches indicate clades also occurring on the full matrix representation parsimony supertree, whereas red branches indicate conflicting clades; branch lengths do not reflect divergence times or amount of sequence divergence. Most of the conflict between the two trees derives from three unusual results from the supermatrix analysis: 1) a paraphyletic *Vulpes *nesting within Canini within Canidae, 2) a paraphyletic Mephitidae and *Ailurus fulgens *nesting within Procyonidae, and 3) a placement of *Eira barbara *away from *Gulo *+ *Martes *and within Lutrinae.

The supertree is also moderately different from a tree derived from a supermatrix analysis of the molecular sequence data (22.9% for 229 species). Many of the differences occur within the different families, with the higher-level relationships of both trees being largely identical (Figure 11). The degree of difference is deceptive, however, with much of the apparent conflict deriving from the supermatrix analysis failing to reconstruct a number of reasonably uncontroversial relationships. For instance, it indicates a paraphyletic (but fully resolved) Procyonidae due to the inclusion of *Ailurus fulgens*, a polyphyletic Mephitidae with the separation of *Conepatus semistriatus*, and a non-monophyletic Canini and Vulpini with the inclusion of a paraphyletic *Vulpes *within Canini (ignoring the placements of the controversial genera *Nyctereutes, Otocyon *and *Urocyon*; see Figure 11). The latter result in particular throws all of Canidae in complete disarray. It also does not appear to be an analysis artifact (that is, a suboptimal topology within canids), given that the likelihood score of the supermatrix tree with the topology of the canid subtree substituted for that in Figure 3 is worse than that of the unconstrained supermatrix tree (-430,541.862576 versus -429,035.506738, respectively). The very low bootstrap values for this part of the supermatrix tree (results not shown) indicate a surprising lack of signal within and/or a high degree of conflict among the sequence data. Interestingly, a weighted supertree analysis of the supermatrix data set as individual gene trees does reconstruct all these relationships properly (that is, in agreement with current phylogenetic opinion), again highlighting the different levels on which the supertree and supermatrix approaches operate (trees versus individual characters, respectively; see [81]) as well as the positive effect of weighting MRP analyses according to some measure of evidential support from the source trees [82,83].

Beyond these differences, a key advantage of the supertree approach remains its ability to use more of the global phylogenetic database for a group and therefore to provide a more complete phylogenetic estimate. Despite the tremendous increase in the amount of available sequence data for the carnivores (248 species as of December 2007; see also [15]), most species remain characterized for few genes (see above) and it is only with the inclusion of literature information that a complete estimate is possible. Much of the latter information could be included in a supermatrix framework; however, it would involve extracting the individual character data from each study and assessing the global data set for homology and redundancy, an exceedingly time-consuming process that is probably unrealistic in most instances (see [84]). Our solution of down-weighting non-independent data sets does not optimally account for any redundancy, but does represent a conservative solution insofar as it assumes complete redundancy among all studies within a data partition. Finally, inclusion of the literature data within a supermatrix context also presents analytical problems in that molecular sequence data are arguably best analyzed within a likelihood framework whereas maximum parsimony is better suited for non-molecular data (despite the existence of models of evolution for such data; for example, [85,86]). Thus, at least one of the two partitions will be analyzed suboptimally through the use of a common optimization criterion needed under a supermatrix framework.

### Macroevolutionary trends

All analyses indicate the presence of significant variation in diversification rate across carnivores. This result contrasts with that for the original supertree, where the lineages-through-time plot was virtually straight [87] and only a handful of lineages possessed more extant descendants than expected for their age [1]. In particular, the general additive model (GAM) analyses show a highly significant temporal variation in rate (χ^2 ^= 40.15, df = 9, *P *< 0.001, adjusted *R*^2 ^= 45.8%, deviance explained = 12.8%) with a model in which the diversification rates trends receiving more support than one in which it steps suddenly (evidence weights = 0.9931 and 0.0069, respectively). Although the value is not significant, the Pybus and Harvey **γ **statistic is positive for the current supertree (0.930, two-tailed *P *= 0.3524), indicating a tendency to more numerous recent speciation events than expected under a pure-birth model.

**Figure 12:**
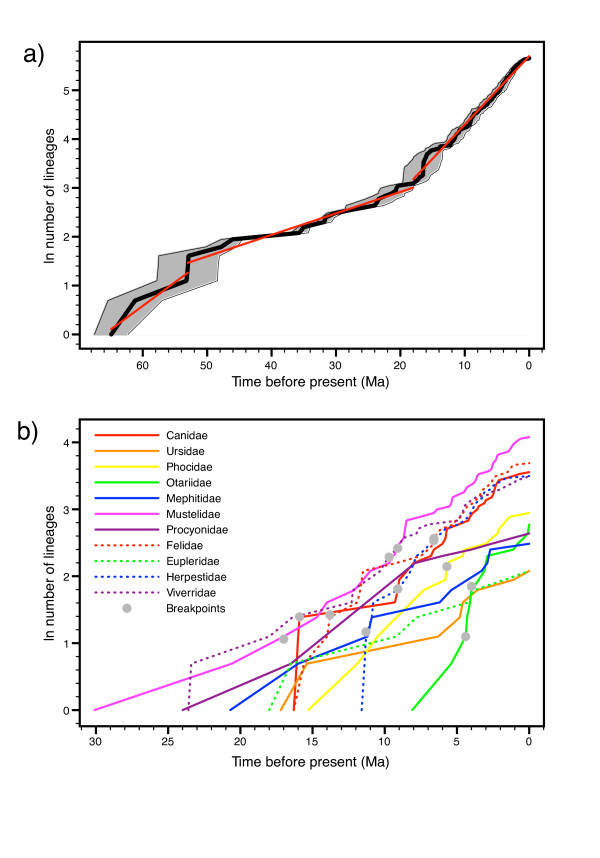
**Lineages-through-time plots for the carnivores (a) as a whole and (b) for the individual families**. In (a), the thick black line indicates the number of lineages through time according to the best divergence time estimates, with the grey area indicating the numbers according to the upper and lower 95% confidence intervals on these dates; the red lines present the slopes for the three different time segments indicated by goodness-of-fit tests. In (b), only the plots according to the best divergence time estimates are presented.

**Figure 13:**
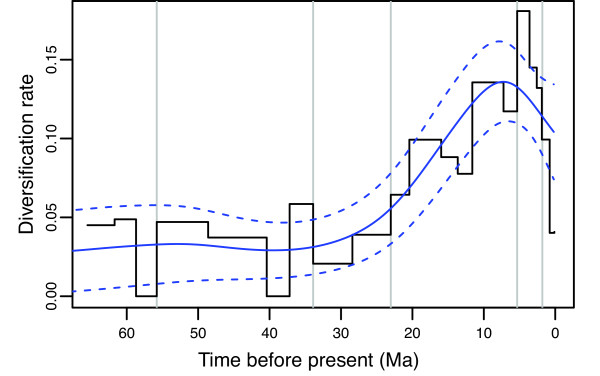
**Net diversification rates through time across all carnivores**. Rates were localized to bins of 0.25 Ma (stepped line) or modeled as a function of time using generalized additive models (solid curve with 95% confidence intervals presented as dashed curves).

Segmented least squares linear regression indicates three clear phases in the lineages-through-time plot of the carnivores, with two periods of slightly higher rates of net diversification flanking an intermediate period ranging from 18.0 to 53.0 Ma ago (Figure 12), with a minimum rate at 39.0 Ma ago (GAM analyses; Figure 13). The intervening period of comparatively low net diversification rates reflects the often extended lag times between the origins of the different families and the basal radiations within the crown groups, and could possibly be the signature of the pattern of competitive subclade replacement known for at least canids and felids. The reality of the breakpoint at 53.0 Ma ago might be undetermined to some degree by the higher stochastic variation present at this time (note the larger CI in Figure 12) resulting from the low number of lineages (T Stadler, personal communication). Thus, it could represent a time when the lineages-through-time plot is essentially 'warming up'. However, it is weakly indicated by the GAM-based diversification analyses, which similarly reflect that many lineages are quickly coming into existence at the base of the carnivore tree (see Figure 2), meaning that a true adaptive radiation at this time cannot be discounted absolutely.

The shift at 18.0 Ma ago corresponds roughly to the period when most of the different families began radiating (see above), leading to a peak in the net diversification rate at about 7.3 Ma ago (GAM analyses; Figure 13). It does not appear to be driven by the obvious hump in the plot at 16.5 Ma ago, which appears to be an artifact traceable to the large basal polytomy within Procyonidae mimicking an adaptation radiation at this time. (Removing the polytomy by pruning all species of *Bassaricyon *and *Bassariscus *from the tree largely removes the hump and leaves the positions of the breakpoints unaffected (results not shown).) By contrast, the TreePar analyses indicate four significant shifts at *P *< 0.01, with three of these shifts (15.8, 16.4 and 16.5 Ma ago) clearly being artifacts deriving from the procyonid polytomy (T Stadler, personal communication).

However, the TreePar analyses also indicate a significant shift at 2.2 Ma ago when the lineages-through-time plot flattens out. This shift, which is in fact the most significant of the four, does not derive from any terminal polytomies (results not shown). It is probably too recent to be an artifact arising from recent cladogenetic events not being recognized as separate species (see [88]), a supposition supported by the combination of the latter part of the lineages-through-time plot for the original supertree also being flat [87] with there having been several subspecies elevated to full species status in the meantime. Instead, the slowdown could be a result of density-dependent speciation (see [89]) or the signature of a recent mass extinction from which carnivores have yet to bounce back (see [87]).

These global analyses, however, disguise a diversity in macroevolutionary patterns across the major carnivore lineages (Figure 12). Whereas Eupleridae, Mephitidae, Procyonidae and Ursidae each present a constant net speciation rate over their entire history, the remaining, generally larger, lineages display at least one inflection point in their lineages-through-time plots. All patterns of change are presented for the latter groups, including general slowdowns (Herpestidae, Phocidae and Viverridae) or speedups (Mustelidae) in the diversification rate moving towards the present, and higher intermediate rates than in the flanking periods (Otariidae) or the reverse (Canidae and Felidae). Many of the lineages display conspicuous jumps in the plots, particularly shortly after the origins of the families, pointing to adaptive radiations. Overall, however, the lack of a consistent pattern of change as well as the inflection points failing to fall into a narrow time window indicate that there were apparently no factors influencing carnivore diversity globally. This is perhaps not unexpected given the worldwide distribution of the order. Instead, explanations for changes in diversification rate need be sought on a group-by-group basis.

That being said, three sets of interesting parallels are observable from the lineages-through-time plots of the individual families. First, all families except Ursidae present negative values of the Pybus and Harvey **γ **statistic, with the values being significant for Herpestidae (-3.510; two-tailed *P *= 0.0004) and, at least not corrected for multiple comparisons, for Procyonidae (-2.020; two-tailed *P *= 0.0433). This is in stark contrast for the positive value observed over the entire order. Second, Canidae, Felidae and Otariidae show nearly identical net diversification rates (0.195 to 0.198) for the last phases in their plots, despite the different times at which the breakpoints occur as well as the disparity among the groups in general (for example, number of species, lifestyle and habitat, degree of relatedness, pattern of diversification). Third, the timings of the last breakpoints are identical between Canidae and Mustelidae (both 9.1 Ma ago) and between Felidae and Herpestidae (both 6.6 Ma ago). In the former case, both families display an increase in rate, whereas in the latter case only Felidae show an increase, with Herpestidae undergoing a marked decrease. It remains an open question as to whether these parallels are merely coincidental or are somehow correlated, either directly (for example, through competition between the groups) or indirectly (for example, as reactions to common biotic or abiotic factors).

Similarly, all but one of the seven whole-tree measures implemented in SymmeTREE (*I_c _*for the most asymmetric resolution of polytomies) indicated significant variation in diversification rates across all carnivores (results not shown; except for *I_c_*, all *P *values were ≤ 0.00153). Application of the shift statistics localized five sister groups with significant (uncorrected *P *< 0.05) and eight others with marginally significant (uncorrected *P *< 0.10) rate variation in at least one shift statistic (Table 4). Among the former five, notable shifts involve *Ailurus fulgens *and *Nandinia binotata *and their much larger sister groups, but not the relic Hyaenidae and Odobenidae as might have been expected. Most of the remaining shifts were localized within individual families and within Mustelidae in particular. In noting that the timings of the shifts often do not accord with the changes in net diversification rate found in the lineages-through-time plots, it must be remembered that SymmeTREE essentially examines for shifts horizontally across sister groups, whereas lineage-through-time plots function vertically through time.

**Table 4:** Local shifts in diversification rate within carnivores.

Node number	Clade size	Nodal depth from root	Clade 1 (number of species)	Clade 2 (number of species)	Δ_1_	Δ_2_	**Slowinski-Guyer **[**91**,**92**]
8	121	1	*Nandinia binotata *(1)	remaining Feliformia (120)	4.66 (0.0052)	4.43 (0.0085)	0.0167 (0.0484)
22	12	9	*Felis manul *(1)	*Prionailurus *+ remaining *Felis *(11)	2.38 (0.0364)	2.30 (0.0364)	0.1818 (0.0364)
134	74	5	*Ailurus fulgens *(1)	Mustelidae + Procyonidae (73)	3.63 (0.0175)	3.33 (0.0221)	0.0274 (0.2063)
137	57	8	*Taxidea taxus *(1)	most remaining Mustelidae (56)	2.90 (0.0279)	2.64 (0.0318)	0.0357 (0.3095)
141	38	12	'Paradoxurinae' + Hemigalinae (11)	Viverrinae + Genettinae (27)	2.22 (0.0497)	2.17 (0.0527)	0.1081 (0.0106)
52	11	5	*Arctogalidia trivirgata *(1)	remaining "Paradoxurinae" + Hemigalinae (10)	2.23 (0.0667)	2.08 (0.0667)	0.2000 (0.0667)
124	130	2	Ursidae (8)	remaining Arctoidea (122)	1.59 (0.0929)	1.42 (0.1150)	0.1240 (0.1399)
144	12	15	*Enhydra lutris *(1)	most remaining Lutrinae (11)	2.27 (0.0727)	2.08 (0.1091)	0.1818 (0.0929)
204	16	6	*Callorhinus ursinus *(1)	remaining Otariidae (15)	2.32 (0.0762)	2.08 (0.0952)	0.1333 (0.1618)
237	35	2	*Urocyon *(2)	remaining Canidae (33)	2.07 (0.0628)	1.91 (0.0773)	0.1176 (0.0501)
239	20	4	*Lycaon *+ *Speothos *(2)	remaining Canini (18)	1.48 (0.1128)	1.32 (0.1369)	0.2105 (0.0792)

Shifts were inferred using SymmeTREE [90]. All nodes that presented significant (*P *< 0.05) or marginally significant (*P *< 0.10) shifts for any of the three statistics are listed.

### Conservation biology

Phylogenetic diversity (PD; [93]) across the entire Carnivora amounts to 2528.3 Ma in total or an average of 8.8 Ma for each of the 286 species. The PD, at least on a per species basis, is divided relatively equally among Feliformia (9.1 Ma per species; 1104.8 Ma in total) and Caniformia (8.4 Ma per species; 1380.8 Ma in total). Among the families, per species PD values range from a minimum of 4.2 Ma for the recent Otariidae radiation to a maximum of 13.8 Ma for Procyonidae. This last value excludes the very small, generally monotypic families from consideration (that is, Ailuridae, Nandiniidae, Odobenidae and Prionodontidae), which generally have much higher values (for example, 53.2 Ma for *Nandinia binotata*), and is itself an overestimate given the large polytomy involving *Bassaricyon*, thereby resulting in overestimated divergence time estimates for most of the species in this genus. Otherwise, the maximum value of 10.8 Ma per species is achieved by Eupleridae. The remaining families have values ranging between 6.0 and 9.0 Ma per species.

There is high overlap in those species showing the highest amounts of PD and evolutionary distinctiveness (ED; [94]) (Table 5), with seven species being in the top 10 for both statistics: *Ailuropoda melanoleuca, Ailurus fulgens, Arctogalidia trivirgata, Nandinia binotata, Odobenus rosmarus, Potos flavus *and *Taxidea taxus*. Most of these species sit isolated at the ends of very long branches (17.2 Ma or more). Species generally showed higher ED scores than higher-level taxa. Only Prionodontidae would be included in the top 10 of all taxa and/or clades (21.19; tied for #8 with *Taxidea taxus*) by virtue of both its constituent species, *Prionodon linsang *and *Prionodon pardicolor*, also ranking among the top 10 ED species. Otherwise, the next highest placed clade is that comprising *Lyncodon patagonicus *and *Mellivora capensis *(12.73, position #35) followed by the genus *Urocyon *(8.99; position #99).

**Table 5:** Summary statistics for the 25 carnivore species falling within the top 10 for any of phylogenetic diversity, evolutionary distinctiveness, or EDGE scores

Species	PD	PD Rank	ED	ED Rank	Red List status	EDGE	EDGE Rank
*Monachus monachus*	9.2	28	11.42	44	CR	5.29	1
*Ailuropoda melanoleuca*	17.2	8	21.24	7	EN	5.18	2
*Urocyon littoralis*	1.1	256	10.09	62	CR	5.18	2
*Monachus schauinslandi*	4.6	93	9.12	91	CR	5.09	4
*Procyon pygmaeus*	2.6	179	8.76	102	CR	5.05	5
*Ailurus fulgens*	35.0	2	35.36	2	VU	4.98	6
*Viverra civettina*	2.6	179	7.61	137	CR	4.93	7
*Lynx pardinus*	2.5	191	6.27	182	CR	4.76	8
*Cynogale bennettii*	10.0	24	12.97	30	EN	4.72	9
*Lycaon pictus*	6.6	58	9.69	74	EN	4.45	10
*Pteronura brasiliensis*	8.7	38	9.66	76	EN	4.45	10
*Tremarctos ornatus*	15.4	16	19.69	9	VU	4.42	13
*Cryptoprocta ferox*	18.0	6	19.11	11	VU	4.39	14
*Nandinia binotata*	53.2	1	53.38	1	LC	4.00	24
*Prionodon linsang*	10.9	22	32.09	3	LC	3.50	49
*Prionodon pardicolor*	10.9	22	32.09	3	LC	3.50	49
*Potos flavus*	24.0	3	24.95	5	LC	3.26	65
*Taxidea taxus*	20.6	4	21.19	8	LC	3.10	69
*Arctogalidia trivirgata*	18.0	6	19.21	10	LC	3.01	72
*Bassaricyon alleni*	16.5	9	18.02	14	LC	2.95	78
*Bassaricyon beddardi*	16.5	9	18.02	14	LC	2.95	78
*Bassaricyon gabbii*	16.5	9	18.02	14	LC	2.95	78
*Bassaricyon lasius*	16.5	9	18.02	14	DD	0.00	239
*Bassaricyon pauli*	16.5	9	18.02	14	DD	0.00	239
*Odobenus rosmarus*	20.4	5	21.40	6	DD	0.00	239

CR: critically endangered; DD: data deficient; ED: evolutionary distinctiveness; EDGE: Evolutionarily Distinct and Globally Endangered; EN: endangered; LC: least concern; PD: phylogenetic diversity; VU: vulnerable.

The top 10 Evolutionarily Distinct and Globally Endangered (EDGE) taxa [94] (Table 5) contain all six carnivore species listed as critically endangered under the 2010 International Union for Conservation of Nature Red List. Five of these top 10 species also fall under the top 100 EDGE mammals [95]: *Ailuropoda melanoleuca, Ailurus fulgens, Monachus monachus, Monachus schauinslandi *and *Viverra civettina*. Indeed, the two pandas were also the only species to rank among the top 10 for all three statistics, highlighting both their evolutionary novelty and precarious conservation position. The new phylogenetic perspective provided here means that two species, *Urocyon littoralis *and *Procyon pygmaeus*, both of which are critically endangered, have higher EDGE values than some of these five and so should be deserving of additional conservation attention and effort.

## Conclusions

Although the carnivore supertree of Bininda-Emonds *et al*. [1] was a reasonably accurate reflection of phylogenetic opinion at the time, the results were still influenced by several methodological limitations. Some of the latter (for example, assumptions of 'uncontested' monophyly) were severe enough to predestine the results. The current study removes all these limitations, thereby enabling an unconstrained estimate of phylogenetic relationships and divergence times within Carnivora. Moreover, compared to the previous study, our analyses make better use of the wealth of molecular sequence data that have been compiled for the order in the meantime. The end result is a nearly fully resolved phylogeny, complete with divergence times.

To the best of our knowledge, both carnivore supertrees remain the only complete phylogenetic estimates for all extant species in the group (according to the prevailing taxonomic lists at the time) using as much of the global phylogenetic database as possible (that is, molecular and morphological). Thus, just as the previous version formed an important foundation for a myriad of studies investigating carnivore ecology, biology and evolution, the same will be true of the new supertree. We briefly provide examples of this in terms of conservation biology as well as macroevolutionary patterns across the group as a whole and within its major lineages. Many other applications are possible and the new version of the carnivore supertree should prove to be as indispensable as the previous one.

## Methods

### Data

The data set underlying the updated version of the carnivore supertree was again derived exclusively from previously published sources. Unlike the 1999 tree, however, literature source trees were used only for data that were not single DNA sequences. For the latter, source trees were instead derived *de novo *using sequence data downloaded from GenBank to provide the largest, most comprehensive individual gene trees possible.

#### Literature source trees

The foundation of this data set lies in the 274 source trees from Bininda-Emonds *et al*. [1], which essentially covered the time period from 1970 to 1995 (with some pre-1970 publications). All source publications were re-reviewed and the source trees electronically saved exactly as they appeared in the original publication in NEXUS format [96] using Mesquite v2.x [97]. (In the original analysis, the MRP matrices were entered directly by hand into the data editor of MacClade v3.x [98].)

This data set was then expanded to cover papers published since 1995 (to August 2008) or those that were missed in the original set of analyses. Literature searches used diverse online databases-Carnivore Ecology & Conservation, Google Scholar, ScienceDirect, Scopus, the online catalog of the University of Jena/Provincial Library of Thuringia, Web of Science, Wiley InterScience and Zoological Record-using the search terms phylogen* or taxonom* or systemat* or cladistic* or clado* or classif* or morpholo* or crani* or bone or character or structu* in combination with the scientific or common names of each carnivore family. Secondary searches excluded publications including the keywords DNA or parasit* or molecul* or prädato* or genet* or mitoch*.

To counteract problems associated with data duplication among source trees and source trees of poor quality (see [29]), all source trees were subjected to the selection protocol outlined in Bininda-Emonds *et al*. [99] and excluded for one or more of the following reasons:

1. A publication date before 1970 so as to favor newer source trees based on more comprehensive data sets and analyzed using robust phylogenetic algorithms.

2. Insufficient information in the paper as to the data source underlying the source trees (for example, [100-105]).

3. Trees where characters were merely mapped onto an existing phylogeny unless it was explicitly mentioned that the characters were entirely congruent with the phylogeny (thereby representing independent support for it).

4. Papers lacking trees entirely and where the text was insufficient to accurately reconstruct the pattern of relationships implied for a source tree.

5. All molecular trees based on DNA sequence data that we were able to obtain separately (see below).

Where a publication contained more than one source tree, all independent source trees were identified using the protocols outlined in [99,106]. For non-independent source trees in the same publication, the preferred tree was the consensus of these trees in the first instance, followed by the most taxonomically complete tree or that explicitly preferred and justified by the authors. If none of these options were available, all source trees were coded and included in the main analysis; no mini-supertrees were made (*contra *[99]).

A total of 114 trees were thus obtained, 86.3% of which were also used by Bininda-Emonds *et al*. [1]. (The much smaller number compared with the original study (274) is because the original, nested supertree analysis meant that the same source tree could simultaneously contribute to more than one supertree analysis.) Of the original source trees, 27 were excluded for the reasons outlined above.

As a final step, the taxonomy of the source trees was standardized to the list of species names found in Wozencraft [14] using the Perl script synonoTree.pl v2.2 [99]. Taxon names that did not belong to crown group Carnivora were deleted outright, although the source trees containing them were held to be rooted (see below). For higher-level taxon names, the name-bearing type species was substituted wherever possible (for example, *Canis lupus *for Canidae); otherwise, the taxon was deleted from the source tree as was also the case for ambiguous names (for example, dog). synonoTree also accounts for species that occur more than once on a given tree through the synonymization process by outputting all possible permutations of the tree with the taxa represented once in all possible positions and combinations; these non-independent trees could later be down-weighted appropriately. Including permutations that arose because of the synonymization process, a total of 241 literature trees were obtained.

#### Source trees from DNA sequence data

To obtain the most taxonomically comprehensive gene trees possible, the GenBank flat file corresponding to non-primate, non-rodent mammals (gbmam.txt, v163.0; December 15, 2007) was downloaded and parsed for all possible genes for carnivoran species using the Perl script GenBankStrip.pl v2.1. Only sequences longer than 200 bp (50 bp for transfer RNA (tRNA) genes) and present for more than 20 GenBank species of Carnivora were included. For any given species and gene, only the 10 longest sequence lengths were retained; each 'length' (for example, 1140 bp for *MT-CYTB*) could be represented by multiple sequences of that length. Homologous outgroup sequences for various species of ungulate (*Bos taurus, Equus caballus*), Chiroptera (any of *Artibeus jamaicensis, Mystacina tuberculata *or *Tadarida brasiliensis*); Primates (*Homo sapiens*) and Rodentia (either *Mus musculus *and/or *Rattus norvegicus*) were later added manually once the final set of genes was determined and aligned.

In performing a study such as this, one is naturally reliant upon the quality of the data in GenBank, which has frequently been called into question (for example, [107]) for reasons ranging from simple sequencing errors to more serious problems including contamination (including unrecognized pseudogene copies), false taxonomic identifications and erroneous annotations (including wrong ortholog assessment). Because all sequence data were processed manually at some point (see below), we are confident that many of these errors were identified. Outstanding problems would include erroneous base-calling or false taxonomic identifications on the part of the original authors; a simple fix for either problem does not exist and recognizing such errors might in fact be impossible in the absence of vouchered specimens and chromatograms.

Sequence data were imported into BioEdit v.7.0.5 [108] and aligned using any of Clustal W [109] (as integrated in BioEdit), transAlign.pl [110] in combination with either Clustal W or MUSCLE [111], or MUSCLE in isolation. All alignments were then improved manually by eye in BioEdit before the outgroup sequences were added and aligned.

Finally, analogous to the source trees from the literature, the gene data sets were standardized to the species-level taxonomy of Wozencraft [14] using the Perl script seqCleaner v1.1. At the same time, seqCleaner also removed flanking regions only present in a minority of species, deleted those sequences possessing > 5% undefined nucleotides (Ns), selected the sequence closest to the consensus sequence for species represented by more than one sequence, and ensured that all sequences overlapped pairwise by a minimum of 100 bp (20 bp for tRNAs).

The final molecular data set (see Additional file 1) comprised 74 genes (43,834 bp), of which 41 were nuclear DNA (nDNA; 28,346 bp), 18 were tRNAs (1,313 bp) and the remaining 15 were other mitochondrial DNA (mtDNA) genes (14,175 bp). The individual data sets were also concatenated into a single supermatrix (*sensu *[11]) using the Perl script seqCat.pl v1.0. In addition, a supermatrix of all mtDNA genes (that is, including the tRNA genes) was also constructed, reflecting the clonal and therefore non-independent inheritance of all genes on the mitochondrial genome.

Phylogenetic analyses of the gene data sets as well as of the supermatrix were performed under a maximum likelihood (ML) framework using RAxML v7.0.4 [112] as directed by the Perl script batchRAXML v1.2. All analyses used the GTRMIX model of evolution except for those data sets with less than 50 species where the GTR + G model was used. The GTRMIX model calculates trees initially using the CAT approximation to the gamma distribution before calculating the final likelihood score under a true gamma approximation. For the supermatrix analyses, the parameters of the model were optimized individually for each gene partition. Searches used the rapid bootstrapping algorithm [113] with 1,000 replicates and trees were rooted using the outgroup sequences as appropriate. Where multiple outgroups were present, it was assumed that the primates and/or rodent sequences comprised the most distant outgroups, followed by the bats and then the ungulates (following [34]). Thereafter, any outgroup taxa were pruned from the gene tree and the respective trees held to be rooted.

### Supertree construction

As for the initial analysis, supertree construction used matrix representation [114,115] to code the topology of the source trees as a series of partial binary characters corresponding to each node on the source tree (0 = not descended from node, 1 = descended from node, ? = species not present on tree). In the current analyses, however, semi-rooted coding [116] was employed, which essentially treats the fictional outgroup taxon as any other species. For trees in which the taxon is present (that is, rooted trees), the outgroup is encoded with 0 s, otherwise it is encoded with ?s (that is, for unrooted trees). All but 14 of the original 114 literature trees were unrooted, whereas 63 of the 74 gene trees were rooted. The supertree matrix was obtained using the Perl script SuperMRP.pl v2.2 [116].

Analysis of the matrix used a weighted parsimony approach in which nodes were weighted in the first instance according to their evidential support as estimated using bootstrap frequencies. Weighting in this fashion has been shown to improve the accuracy of MRP supertree construction in simulation [82,83], which even when unweighted shows high accuracy, comparable to pure supermatrix-based analyses [83,117]. However, because this procedure requires that equivalent information is present for all nodes [118], nodes lacking this information (that is, all literature based trees) were weighted effectively neutrally according to the average bootstrap support over all nodes possessing this information (as calculated by SuperMRP.pl).

In the second instance, nodes were down-weighted according to the non-independence of the source trees. Literature source trees were grouped into one of 12 subjective categories according to the source data type (karyotype, scent gland, isozyme, general morphology, mixed morphology and molecular, DNA-DNA hybridization, immunological distances, mixed molecular, satellite DNA, hemoglobin, crystalline and vocalization; see Additional file 4) and the corresponding nodes were down-weighted according to the number of source trees in each set. Finally, nodes were further down-weighted according to any non-independence among the corresponding source trees because they represented either one of many equally valid, non-independent trees in a paper or a permutation from the synonoTree synonymization process. The final weight for any node, therefore, was the bootstrap support for that node multiplied by one or both corrections for data non-independence. Source trees were not otherwise weighted according to the perceived quality of the data or the analyses on which they were based.

Finally, the tree corresponding to the taxonomy of Wozencraft [14] was added as a seed tree to guide the analysis and ensure sufficient overlap between all pairs of species (see [83]). To minimize its impact on the final topology, however, the nodes on the seed tree were down-weighted by a factor of 10 relative to the minimum weight assigned to any other node, meaning that the relationships present in the seed tree could be easily overruled by any real source tree. Exclusion of the seed tree results in only minimal differences compared to the full analysis (normalized partition metric = 7.8%), mostly due to a loss of resolution in some parts of the tree (79.9% versus 93.3% for the full supertree).

In total, five different supertree analyses using different combinations of the source trees were performed: all 241 individual source trees from the literature and all 74 individual gene trees; all individual source trees from the literature, the supermatrix mtDNA tree and all 41 individual nDNA gene trees; all individual source trees from the literature and the supermatrix tree of all individual gene data sets; all individual gene trees only; and all individual source trees from the literature only. All five analyses included differential weighting for nodal support and data non-independence where applicable as well as the seed tree of Wozencraft [14].

A parsimony ratchet [119] was used in the MRP analyses via PAUP* v.4.0b10 [120]. The ratchet employed 50 batches of 200 iterative reweighting steps, followed by a conventional heuristic search (tree bisection and reconnection branch swapping, 10,500 maxtrees) using the ratchet trees as a starting point. The final supertree was the strict consensus of all equally parsimonious solutions found. The instruction file for the ratchet searches was obtained using the Perl script perlRat.pl v1.1.

Support for the nodes in all supertrees was estimates using rQS [30,121] as implemented in qualiTree v2.0b [30]. This measure compares the topology of the supertree to that of the set of source trees (with both pruned to possess identical taxon sets), classifying the latter as either supporting (+1), conflicting with (-1) or being equivocal (+0) to a given node. rQS is then the average of the non-zero (that is, informative) values contributing to it and can range from +1 (universal support) to -1 (universal conflict). Typical measures lie around 0 such that even slightly positive values of rQS tend to indicate reasonable support.

In this study, only the first supertree, which represents our preferred hypothesis, is presented and the pattern of relationships on it discussed explicitly. The remaining supertrees and supermatrix tree (Additional file 3) are instead only compared generally to the main supertree, highlighting key areas of disagreement. Differences between all pairs of trees (pruned to the same taxon set as needed) were quantified using a normalized partition metric [79,80] as implemented in the Perl script partitionMetric v1.2.2. The first supertree and associated data matrix can be downloaded from TreeBASE at http://purl.org/phylo/treebase/phylows/study/TB2:S11954; dated versions of the supertree are also available in Additional file 5; all remaining trees are available in Additional file 3.

### Divergence time estimation

To estimate divergence times throughout the order, a local molecular clock analysis was used as implemented in the Perl script relDate.pl v3.0b [34]. The relDate method, which originates with Purvis [122], fits a set of DNA sequence data to the topology of the reference tree under the optimal model of evolution in a ML framework to establish the heights of each node relative to some ancestral and/or daughter node. These relative heights are then calibrated against one or more points to derive absolute divergence time estimates. The procedure is performed individually for each gene in the data set, with the final date estimate for a given node being the median of all genetic or fossil estimates or the youngest fossil estimate alone, should it be older than the median value. Any negative branch lengths are corrected in a subsequent step as is the estimation of divergence times for nodes missing such values according to a pure-birth model based on relative clade sizes (see [122]). The distribution of date estimates across the genetic and fossil partitions also enables estimation of the upper and lower 95% CI for each nodal date. Further details regarding the relDate procedure can be found in the Supplementary Information of Bininda-Emonds *et al*. [37].

Divergence dates were estimated for the main, preferred supertree only using the same gene data sets used to build the supertree. All dates were calculated as the weighted average of the relative dates from the relevant gene trees, with the contribution of a given gene tree to the node being weighted according to the bootstrap support of that node on the gene tree. As such, nodes on gene trees that support the supertree topology more strongly (that is, the underlying data set agreed with the supertree topology) had a proportionately greater influence on the divergence time estimate for that node (OBE, in preparation).

Absolute divergence dates were obtained by calibrating against 12 fossil estimates or ranges from the literature (Table 1). The calibration points were chosen due to their use in previous studies [33,37] and were restricted to the major clades within and outside Carnivora, where the assignments of the fossils are arguably the most robust. For those calibration points from Bininda-Emonds *et al*. [37] at least, one requirement was that any fossils must be associated with their clade in at least one cladistic study, such that at least one synapomorphy links the fossil with the relevant crown group. For calibrations in the form of ranges, the midpoint of the range was used as the actual calibration with the extremes of the range being used as age constraints.

The optimal model of evolution for each gene data set using the supertree as a reference tree was determined using ModelTEST [123] in combination with PAUP* under an Akaike information criterion (AIC) as implemented in the Perl script autoMT.pl v1.0. The applicability of a molecular clock was tested using a likelihood ratio test with a nominal *P *of 0.05 corrected for multiple comparisons using a sequential Holm-Bonferroni correction [124]. Thereafter, the sequence data were fitted to the topology of the main supertree under the optimal evolutionary model (see Additional file 6; and [125,126]) using PAUP* and the Perl script seqFitter.pl v1.0. Gene trees for those data sets for which the molecular clock hypothesis could not be rejected were regarded as being rooted; all other gene trees were unrooted. Using the gene trees, the final date estimates (including corrections for any negative branch lengths and estimation of missing dates) and the upper and lower 95% CI for them were obtained using relDate.pl v3.0b.

### Macroevolutionary analyses

Patterns of diversification within carnivores were visualized using lineages-through-time plots [127], both for the order as a whole as well as for the individual families, and quantified using the **γ **statistic of Pybus and Harvey [128], which compares the relative heights of the internal nodes to those expected under a pure-birth model. Shifts in the net diversification rate were inferred using three methods: segmented least squares regression; localized estimation of net diversification rate through survival analysis (using bin widths of 0.25 Ma) followed by modeling the rate as a function of time using GAMs (see [37]); and using TreePar v2.1.2 [129]. For the first method, the goodness-of-fit tests based on the AIC were used to select the best regression model allowing up to two breakpoints spread at all points along the lineages-through-time plots. TreePar analyses were conducted for up to four shifts, with the number of significant shifts being determined at *P *< 0.01 using a likelihood ratio test.

In addition, the whole-tree measures implemented in SymmeTREE [90] were used to identify if variation in diversification rate was present across carnivores independent of divergence time information, with the various shift statistics used to pinpoint sister groups showing significantly different rates. For these analyses, the default settings of the program were used, but with polytomies randomly resolved according to a TSS-ERM with 1,000,000 permutations.

## Competing interests

The authors declare that they have no competing interests.

## Authors' contributions

KN and OBE designed the research and wrote the paper. In addition, KN collected and processed most of the literature and all the GenBank data and performed the supertree analyses, whereas OBE performed the dating and macroevolutionary analyses. Both authors read and approved the final manuscript.

## Supplementary Material

Additional file 1 **Summary of all source data used to build the carnivore supertree**. In the accompanying electronic file, a filled-in cell (x for literature sources including Wozencraft [14], GenBank accession number for the gene trees) indicates the data that were available for that species for that source tree. More information for the literature trees can be found in Additional file 4. Click here for file

Additional file 2 **Summary statistics for the nodes on the carnivore supertree**. rQS values are for informative source trees only and the numbers of source trees that supported, contradicted or contained no information with respect to a given node on the supertree are listed. Click here for file

Additional file 3 **Estimates of carnivore phylogeny using different analytical methods and/or variations on the base data set**. The text file contains all additional trees constructed from the literature source trees and/or gene trees or gene data and referenced to in Table 3. The trees can be viewed using Mesquite [97] or a tree viewer like TreeView X [131] or FigTree [132], among others. Click here for file

Additional file 4 **Summary of literature source trees**. Identities of the literature studies, including the exact data source and category into which the source tree was placed. Click here for file

Additional file 5 **The carnivore supertree with divergence time estimates**. The text file contains four topologically identical versions of the carnivore supertree. The first three differ in their branch lengths and reflect the best estimates of the divergence times and the lower and upper 95% CIs on these estimates. The final tree combines these three sets of dates in a single FigTree-specific [132] format using node bars to display the CIs; it might need to be deleted when viewing the file in an application other than FigTree. Otherwise, the trees can be viewed using Mesquite [97] or a tree viewer like TreeView X [131]. Click here for file

Additional file 6 **Optimal models of evolution for the 74 gene data sets for the relDate analyses**. Analyses used autoMT.pl in conjunction with PhyML ([125,126]; nonclock analyses) and PAUP* (clock analyses). An asterisk behind the likelihood ratio test *P *value indicates that the data set did not evolve according to a strict molecular clock at the 0.05 level (corrected for multiple comparisons). Click here for file
