# Supplementary material for: Updating the evolutionary history of Carnivora (Mammalia): a new species-level supertree complete with divergence time estimates
Source: BMC Biol. 2012 Feb 27;10:12. doi: 10.1186/1741-7007-10-12 (PMC3307490; doi:10.1186/1741-7007-10-12)
Supplement: Additional file 4 — Summary of literature source trees. Identities of the literature studies, including the exact data source and category into which the source tree was placed. [file 1741-7007-10-12-S4.PDF]

Additional file 4

Identities of the literature studies, including the exact data source and category into which the source tree was placed.

Study ID	Reference	Source of data	Source tree ID	Category
CJC_pr_2	[1]	Fig. 14	CJC_pr_2_1	morphology
CJC_pr_5	[2]	Fig. 9; names from Table XIII	CJC_pr_5_1	DNA hybridization
CJC_pr_6	[3]	Fig. 3	CJC_pr_6_1	morphology
CJC_pr_9	[4]	Fig. 8a	CJC_pr_9_1	morphology
CJC_pr_10	[5]	Figs. 26–28	CJC_pr_10_1	morphology
CJC_pr_11	[6]	Fig. 2	CJC_pr_11_1	morphology
CJC_pr_12	[7]	Text	CJC_pr_12_1	karyotype
CJC_pr_13	[8]	Text	CJC_pr_13_1	morphology
CJC_pr_14	[9]	Text	CJC_pr_14_1	morphology
CJC_pr_15	[10]	Fig. 1	CJC_pr_15_1	morphology
CJC_pr_16	[11]	Fig. 1	CJC_pr_16_1	isozymes
CJC_pr_17	[12]	Fig. 2	CJC_pr_17_1	morphology
CJC_pr_18	[13]	Fig. 2	CJC_pr_18_1	mixed data
CJC_pr_19	[14]	Fig. 2	CJC_pr_19_1	karyotype
CJC_pr_22	[15]	Fig. 3b	CJC_pr_22_3	restriction maps
		Fig. 3a	CJC_pr_22_4	restriction maps

Study ID	Reference	Source of data	Source tree ID	Category
CJC_pr_23	[16]	Figs 4–8	CJC_pr_23_1	scent compounds
CJC_pr_28	[17]	Fig. 3	CJC_pr_28_1	karyotype
CJC_pr_29	[18]	Table 1	CJC_pr_29_1	morphology
CJC_pr_30	[19]	Text, pg. 38	CJC_pr_30_1	morphology
CJC_pr_33	[20]	Fig. 7	CJC_pr_33_1	karyotype
CJC_pr_34	[21]	Fig. 4	CJC_pr_34_1	karyotype
CJC_pr_35	[22]	Text, pg. 147	CJC_pr_35_1	morphology
CJC_pr_36	[23]	Text, pg. 248	CJC_pr_36_1	morphology
CJC_pr_37	[24]	Text, pg. 257	CJC_pr_37_1	morphology
CJC_pr_38	[25]	Text, pg. 37	CJC_pr_38_1	morphology
CJC_pr_39	[26]	Fig. 1	CJC_pr_39_1	isozymes
CJC_pr_42	[27]	Fig. 1	CJC_pr_42_1	karyotype
CJC_pr_44	[28]	Fig. 23	CJC_pr_44_1	morphology
CJC_pr_45	[29]	Fig. 1	CJC_pr_45_1	morphology
CJC_pr_46	[30]	Fig. 3a	CJC_pr_46_1	restriction maps
		Fig. 3b	CJC_pr_46_2	restriction maps
CJC_pr_47	[31]	Fig. 6	CJC_pr_47_1	morphology
CJC_pr_49	[32]	Fig. 6	CJC_pr_49_1	morphology
CJC_pr_51	[33]	Fig. 1	CJC_pr_51_1	mixed data
CJC_pr_52	[34]	Fig. 1	CJC_pr_52_1	immunology
CJC_pr_53	[35]	Fig. 52	CJC_pr_53_1	morphology

Study ID	Reference	Source of data	Source tree ID	Category
CJC_pr_54	[36]	Text pg.154f.	CJC_pr_54_1	morphology
CJC_pr_56	[37]	Text pg. 101	CJC_pr_56_1	morphology
CJC_pr_57	[38]	Fig. 3.2	CJC_pr_57_1	isozymes
		Fig. 3.2	CJC_pr_57_2	isozymes
CJC_pr_59	[39]	Fig. 4	CJC_pr_59_1	isozymes
CJC_pr_60	[40]	Fig. 1	CJC_pr_60_1	morphology
CJC_pr_62	[41]	Text pg. 402	CJC_pr_62_1	restriction maps
CJC_pr_63	[42]	Fig. 4	CJC_pr_63_1	karyotype
CJC_pr_64	[43]	Table II	CJC_pr_64_1	karyotype
CJC_pr_65	[44]	Fig. 5.9	CJC_pr_65_1	mixed data
CJC_pr_67	[45]	Fig. 4	CJC_pr_67_1	morphology
CJC_pr_68	[31]	Text	CJC_pr_68_1	morphology
KD_pr_1	[46]	Fig. 4	KD_pr_1_1	morphology
		Fig. 5	KD_pr_1_2	morphology
KD_pr_2	[47]	Fig. 3	KD_pr_2_1	morphology
		Fig. 4	KD_pr_2_2	morphology
		Fig. 5	KD_pr_2_3	morphology
KD_pr_3	[48]	Fig. 5	KD_pr_3_1	mixed data
KD_pr_4	[49]	Fig. 5	KD_pr_4_1	morphology
KD_pr_5	[50]	Fig. 8	KD_pr_5_1	morphology
		Fig. 9	KD_pr_5_2	morphology
		Fig. 10	KD_pr_5_3	morphology
		Fig. 10	KD_pr_5_4	morphology

Study ID	Reference	Source of data	Source tree ID	Category
KD_pr_7	[51]	Fig. 1a	KD_pr_7_1	morphology
		Fig. 1b	KD_pr_7_2	morphology
		Fig. 2	KD_pr_7_3	morphology
		Fig. 3	KD_pr_7_4	morphology
KD_pr_8	[52]	Fig. 2	KD_pr_8_1	morphology
KD_pr_9	[53]	Fig. 3	KD_pr_9_1	mixed data
KD_pr_10	[54]	Fig. 10	KD_pr_10_1	mixed data
KD_pr_11	[55]	Fig. 2.8	KD_pr_11_1	mixed data
KD_pr_12	[56]	Fig. 4a	KD_pr_12_1	morphology
		Fig. 4b	KD_pr_12_2	morphology
KD_pr_14	[57]	Fig. 9	KD_pr_14_1	morphology
KD_pr_15	[58]	Fig. 1	KD_pr_15_1	immunology
KD_pr_16	[59]	Fig. 2	KD_pr_16_1	immunology
KD_pr_17	[60]	Fig. 5	KD_pr_17_1	morphology
KD_pr_18	[61]	Fig. 1	KD_pr_18_1	mixed data
KD_pr_19	[62]	Fig. 385	KD_pr_19_1	morphology
KD_pr_20	[63]	Fig. 2	KD_pr_20_1	morphology
KD_pr_21	[64]	Fig. 3	KD_pr_21_1	morphology
KD_pr_22	[65]	Fig. 6	KD_pr_22_1	morphology
KD_pr_23	[66]	Fig. 9	KD_pr_23_1	morphology
KD_pr_26	[67]	Fig. 7	KD_pr_26_1	mixed data
KD_pr_27	[68]	Fig. 11	KD_pr_27_1	immunology
KD_pr_28	[69]	Fig. 6	KD_pr_28_1	mixed molecular

Study ID	Reference	Source of data	Source tree ID	Category
KD_pr_29	[70]	Fig. 12	KD_pr_29_1	morphology
KD_pr_30	[71]	Fig. 1	KD_pr_30_1	morphology
KD_pr_31	[72]	Fig. 17.4	KD_pr_31_1	morphology
KD_pr_32	[73]	Fig. 18.2	KD_pr_32_1	morphology
KD_pr_33	[74]	Fig. 7	KD_pr_33_1	morphology
KD_pr_34	[75]	Fig. 5	KD_pr_34_1	morphology
		Fig. 8	KD_pr_34_2	morphology
KD_pr_35	[76]	Fig. 1	KD_pr_35_1	immunology
		Fig. 2	KD_pr_35_2	immunology
		Fig. 3	KD_pr_35_3	immunology
KD_pr_36	[77]	Fig. 4.1	KD_pr_36_1	morphology
		Fig. 4.1	KD_pr_36_2	morphology
		Fig. 4.1	KD_pr_36_3	morphology
KD_pr_37	[78]	Fig. 27	KD_pr_37_1	karyotype
KD_pr_38	[79]	Fig. 4.2	KD_pr_38_1	morphology
KD_pr_39	[80]	Fig. 42a	KD_pr_39_1	karyotype
		Fig. 42b	KD_pr_39_2	karyotype
KD_pr_40	[81]	Fig. 4	KD_pr_40_1	DNA hybridization
KD_pr_41	[82]	Fig. 4	KD_pr_41_1	immunology
		Fig. 5	KD_pr_41_2	immunology
KD_pr_42	[83]	Figs. 4–8, 11, 13	KD_pr_42_1	morphology
KD_pr_43	[84]	Fig. 6	KD_pr_43_1	morphology
KD_pr_44	[85]	Figs. 1, 5, and 8	KD_pr_44_1	morphology

Study ID	Reference	Source of data	Source tree ID	Category
KD_pr_45	[86]	Fig. 2a	KD_pr_45_1	isozymes
		Fig. 2b	KD_pr_45_2	isozymes
		Fig. 3a	KD_pr_45_3	isozymes
		Fig. 3b	KD_pr_45_4	isozymes
KD_pr_46	[87]	Fig. 3a	KD_pr_46_1	isozymes
		Fig. 3b	KD_pr_46_2	isozymes
		Fig. 3c	KD_pr_46_3	isozymes
		Fig. 4a	KD_pr_46_4	isozymes
		Fig. 4b	KD_pr_46_5	isozymes
		Fig. 4c	KD_pr_46_6	isozymes
		Fig. 5a	KD_pr_46_7	isozymes
		Fig. 5b	KD_pr_46_8	isozymes
KD_pr_47	[88]	Fig. 4a	KD_pr_47_1	isozymes
		Fig. 4b	KD_pr_47_2	isozymes
KD_pr_48	[89]	Fig. 2	KD_pr_48_1	morphology
KD_pr_49	[90]	Fig. 4	KD_pr_49_1	mixed data
KD_pr_50	[91]	Text	KD_pr_50_1	hemoglobin
KD_pr_51	[92]	Text	KD_pr_51_1	morphology
KD_pr_52	[93]	Text	KD_pr_52_1	DNA hybridization
KD_pr_53	[94]	Text	KD_pr_53_1	morphology
KD_pr_54	[95]	Text pp. 239–240	KD_pr_54_1	karyotype
KD_pr_55	[96]	Fig. 29	KD_pr_55_1	morphology
KD_pr_56	[97]	Fig. 1a	KD_pr_56_1	immunology

Study ID	Reference	Source of data	Source tree ID	Category
		Fig 1b	KD_pr_56_2	immunology
		Fig. 1c	KD_pr_56_3	immunology
		Fig. 2a	KD_pr_56_4	isozymes
		Fig. 2b	KD_pr_56_5	isozymes
KD_pr_58	[98]	Text	KD_pr_58_1	morphology
KD_pr_59	[99]	Text	KD_pr_59_1	morphology
KD_pr_61	[100]	Fig. 3	KD_pr_61_1	karyotype
KD_pr_63	[101]	Fig. 27	KD_pr_63_1	morphology
KD_pr_64	[102]	Fig. 12	KD_pr_64_1	morphology
KD_pr_65	[103]	Fig. 6	KD_pr_65_1	morphology
KD_pr_66	[104]	Fig. 51	KD_pr_66_1	morphology
KD_pr_67	[105]	Fig. 6a	KD_pr_67_1	isozymes
KD_pr_68	[106]	Fig. 2	KD_pr_68_1	hemoglobin
KD_pr_72	[107]	Fig. 1a	KD_pr_72_1	isozymes
		Fig. 2a	KD_pr_72_2	isozymes
		Fig. 3a	KD_pr_72_3	isozymes
KD_pr_76	[108]	Fig. 1	KD_pr_76_1	alpha-crystallin
KD_pr_77	[109]	Fig. 2b	KD_pr_77_1	alpha-crystallin
KD_pr_78	[110]	Fig. 18	KD_pr_78_1	karyotype
KD_pr_79	[111]	Fig. 1	KD_pr_79_1	morphology
KD_pr_80	[112]	Fig. 2	KD_pr_80_1	morphology
KD_pr_81	[113]	Fig. 2	KD_pr_81_1	hemoglobin

Study ID	Reference	Source of data	Source tree ID	Category
KD_pr_84	[114]	Fig. 34a	KD_pr_84_1	morphology
		Fig. 34b	KD_pr_84_2	morphology
		Fig. 34c	KD_pr_84_3	morphology
KD_pr_86	[115]	Text pg. 48	KD_pr_86_1	karyotype
KD_pr_87	[116]	Text pp. 389–390	KD_pr_87_1	morphology
KD_pr_88	[117]	Text pp. 142–144	KD_pr_88_1	morphology
KD_pr_89	[118]	Text pg. 328	KD_pr_89_1	vocalization
KD_pr_91	[119]	Text pg. 28	KD_pr_91_1	hemoglobin
KD_pr_93	[120]	Text pp. 203–205	KD_pr_93_1	morphology
KD_pr_95	[121]	Text pg. 322	KD_pr_95_1	morphology
KD_pr_96	[122]	Fig. 2	KD_pr_96_1	morphology
		Fig. 6	KD_pr_96_2	morphology
		Fig. 10	KD_pr_96_3	morphology
		Fig. 11	KD_pr_96_4	morphology
		Fig. 15	KD_pr_96_5	morphology
		Fig. 19	KD_pr_96_6	morphology
KD_pr_97	[123]	Fig. 3	KD_pr_97_1	morphology
		Fig.4	KD_pr_97_2	morphology
		Fig. 5	KD_pr_97_3	morphology
KD_pr_98	[124]	Fig. 1	KD_pr_98_1	isozymes
KD_pr_99	[125]	Text	KD_pr_99_1	vocalization
KD_pr_101	[126]	Text	KD_pr_101_1	immunology
KD_pr_102	[127]	Text	KD_pr_102_1	morphology
KD_pr_103	[128]	Fig. 11	KD_pr_103_1	mixed data

Study ID	Reference	Source of data	Source tree ID	Category
		Fig. 12	KD_pr_103_2	mixed data
KD_pr_104	[129]	Fig. 4.1	KD_pr_104_1	mixed data
KD_pr_107	[130]	Text	KD_pr_107_1	karyotype
KD_pr_109	[131]	Text	KD_pr_109_1	satellite DNA
KD_pr_111	[132]	Fig. 1	KD_pr_111_1	hemoglobin
		Fig. 2	KD_pr_111_2	hemoglobin
KD_pr_113	[133]	Fig. 1	KD_pr_113_1	hemoglobin
KD_pr_114	[134]	Fig. 1	KD_pr_114_1	morphology
KD_pr_115	[135]	Text	KD_pr_115_1	morphology
KD_pr_116	[136]	Fig. 2	KD_pr_116_1	isozymes
KD_pr_118	[137]	Fig. 3	KD_pr_118_1	karyotype
OBE_pr_1	[138]	Fig. 5b	OBE_pr_1_1	morphology
OBE_pr_2	[139]	Fig. 2e	OBE_pr_2_1	scent compounds
OBE_pr_3		Fig. 17.5	OBE_pr_3_1	DNA hybridization
OBE_pr_4	[140]	Fig. 1	OBE_pr_4_1	DNA hybridization
		Fig. 2	OBE_pr_4_2	isozymes
		Fig. 3	OBE_pr_4_3	immunology
OBE_pr_5	[141]	Fig. 7	OBE_pr_5_1	morphology
		Fig. 8	OBE_pr_5_2	morphology

References

1. Baskin JA: **Tertiary Procyonidae (Mammalia: Carnivora) of North America.** *J Vertebr Paleontol* 1982, **2:**71–93.
2. Benveniste RE: **The contributions of retroviruses to the study of mammalian evolution.** In *Molecular evolutionary genetics.* Edited by MacIntyre RJ. New York: Plenum Press; 1985: 359–417
3. Baskin JA: **Comments on New World Tertiary Procyonidae (Mammalia: Carnivora).** *J Vertebr Paleontol* 1989, **9:**110–117.
4. Clutton-Brock J, Corbet GB, Hills M: **A review of the family Canidae, with a classification by numerical methods.** *Bulletin of the British Museum of Natural History* 1976, **29:**120–199.
5. Berta A: *Quaternary evolution and biogeography of the large South American Canidae (Mammalia: Carnivora).* Berkeley and Los Angeles: University of California Press; 1981.
6. Berta A: **Origin, diversification, and zoogeography of the South American Canidae.** *Fieldiana Zool* 1987, **39:**455–471.
7. Anbinder EM: **Karyological similarity of sea otters and other mustelids (Carnivora: Mustelidae).** *Biol Morya (Vladivost)* 1976, **3:**78–79.
8. Crawford-Cabral J: **A new classification of the genets.** *African Small Mammal Newsletter* 1981, **6:**8–10.
9. Crawford-Cabral J: **The classification of the genets (Carnivora, Viverridae, genus *Genetta*).** *Bol Soc Port Cienc Nat* 1982 [intended 1980-1981], **20:**97–114.
10. Ferguson SH, Virgl JA, Larivière S: **Evolution of delayed implantation and associated grade shifts in life history traits of North American carnivores.** *Ecoscience* 1996, **3:**7–17.

11. Dragoo JW, Bradley RD, Honeycutt RL, Templeton JW: **Phylogenetic relationships among the skunks: a molecular perspective.** *J Mamm Evol* 1993, **1:**255–267.
12. Decker DM, Wozencraft WC: **Phylogenetic analysis of Recent procyonid genera.** *J Mammal* 1991, **72:**42–55.
13. Dragoo JW, Honeycutt RL: **Systematics of mustelid-like carnivores.** *J Mammal* 1997, **78:**426–443.
14. Dutrillaux B, Couturier J, Chauvier G: **Notes et discussions sur "Édentes", Carnivores, "Pinnipèdes" et leurs parasites. 3. - Les Pinnipèdes, monophylétiques, sont issus de Procyonidae ancestraux, et non d'Ursidae ni de Mustelidae.** *Mém Mus Natl Hist Nat Sér A Zool* 1982, **123:**141–143.
15. Geffen E, Mercure A, Girman DJ, Macdonald DW, Wayne RK: **Phylogenetic relationships of the fox-like canids: mitochondrial DNA restriction fragment, site and cytochrome *b* sequence analyses.** *J Zool* 1992, **228:**27–39.
16. Decker DM: **The utility of chemical signals as characters in phylogenetic studies: examples from the Felidae.** *Ph.D. dissertation.* University of Knoxville, Tennessee, 1996.
17. Graphodatsky AS, Volobuev VT, Ternovsky DV, Radjabli SI: **G-banding of the chromosomes in seven species of Mustelidae (Carnivora).** *Zool Zh* 1976, **55:**1704–1709.
18. Davis JA: **A classification of the otters.** In *Otters — Proceedings of the first working meeting of the otter specialist group of the survival service commission.* Edited by Duplaix N: IUCN (International Union for

Conservation of Nature and Natural Resources) Publication. New Series; 1978: 14–33

19. Grove C: **Cranial and dental characteristics in the systematics of Old World Felidae.** *Carnivore* 1982, **5:**28–39.
20. Wayne RK, Nash WG, O'Brien SJ: **Chromosomal evolution of the Canidae: I. Species with high diploid numbers.** *Cytogenet Cell Genet* 1987, **44:**123–133.
21. Wayne RK, Nash WG, O'Brien SJ: **Chromosomal evolution of the Canidae: II. Divergence from the primitive carnivoran karyotype.** *Cytogenet Cell Genet* 1987, **44:**134–141.
22. Stains HJ: **Comparative study of the calcanea of members of the Ursdiae and Procyonidae.** *Bull S Calif Acad Sci* 1973, **72:**137–148.
23. Stains HJ: **Calcanea of members of the Mustelidae. Part I, Mustelinae.** *Bull S Calif Acad Sci* 1976, **75:**237–248.
24. Stains HJ: **Calcanea of members of the Mustelidae. Part II, Mellivorinae, Melinae, Mephitinae, and Lutrinae.** *Bull S Calif Acad Sci* 1976, **75:**249–257.
25. Stains HJ: **Calcanea of members of the Viverridae.** *Bull S Calif Acad Sci* 1983, **82:**17–38.
26. Simonsen V: **Electrophoretic variation in large mammals. II. The red fox, *Vulpes vulpes*, the stoat, *Mustela erminea*, the weasel, *Mustela nivalis*, the pole cat, *Mustela putorius*, the pine marten, *Martes martes*, the beech marten, *Martes foina*, and the badger, *Meles meles*.** *Hereditas* 1982, **96:**299–305.
27. Král B, Zima J: **Karyosystematika celedi Felidae.** *Gazella* 1980, **2/3:**45–53.

28. Youngman PM: **Distribution and systematics of the European mink *Mustela lutreola* Linnaeus 1761.** *Acta Zool Fenn* 1982, **166:**1–48.
29. Hemmer H: **Die Evolution der Pantherkatzen. Modell zur Überprüfung der Brauchbarkeit der HENNIGschen Prinzipien der phylogenetischen Systematik für wirbeltierpaläontologische Studien.** *Paläontolog Z* 1981, **55:**109–116.
30. Hosoda T, Suzuki H, Yamada T, Tsuchiya K: **Restriction site polymorphism in the ribosomal DNA of eight species of Canidae and Mustelidae.** *Cytologia* 1993, **58:**223–230.
31. Long CA: **Provisional classification and evolution of the badgers.** *Proc Woldwide Furbearer Conf* 1981, **1:**55–85.
32. Mazza P, Rustioni M: **On the phylogeny of Eurasian bears.** *Palaeontographica Abteilung A Palaeozoologie Stratigraphie* 1994, **230:**1–38.
33. Mattern MY, McLennan DA: **Phylogeny and speciation of felids.** *Cladistics* 2000, **16:**232–253.
34. Schreiber A, Eulenberger K, Bauer K: **Immunogenetic evidence for the phylogenetic sister group relationship of dogs and bears (Mammalia, Carnivora: Canidae and Ursidae): a comparative determinant analysis of carnivoran albumin, C3 complement and immunoglobulin μ-chain.** *Exp Clin Immunogenet* 1998, **15:**154–170.
35. Salles LO: **Felid phylogenetics: extant taxa and skull morphology (Felidae, Aeluroidae).** *Am Mus Novit* 1992, **3047:**1–67.

36. Stains HJ: **Distribution and taxonomy of the Canidae.** In *The wild canids: their systematics, behavioral ecology and evolution.* Edited by Fox MW. New York: Van Nostrand Reinhold Company; 1975: 3–26
37. Peters G, Hast MH: **Hyoid structure, laryngeal anatomy, and vocalization in felids (Mammalia: Carnivora: Felidae).** *Z Säugetierkd* 1993, **59:**87–104.
38. O'Brien SJ, Martenson JS, Eichelberger MA, Thorne ET, Wright F: **Genetic variation and molecular systematics of the black-footed ferret.** In *Conservation biology and the black-footed ferret.* Edited by Seal US, Thorne ET, Bogan MA, Anderson SH. New Haven, Connecticut: Yale University Press; 1989: 21–33
39. O'Brien SJ, Collier GE, Benveniste RE, Nash WG, Newman AK, Simonson JM, Eichelberger MA, Seal US, Janssen D, Bush M, Wildt DE: **Setting the molecular clock in Felidae: the great cats, *Panthera*.** In *Tigers of the world: biology, biopolitics, management and conservation of an endangered species.* Edited by Tilson RL, Seal US. Parkridge, New Jersey: Noyes Publications; 1987: 10–27
40. de Muizon C: **Les relations phylogénétiques des Lutrinae (Mustelidae, Mammalia).** *Géobios Mém Spéc* 1982, **6:**259–277.
41. Suzuki H, Hosoda T, Sakurai S, Tsuchiya K, Munechika I, Korablev VP: **Phylogenetic relationship between the Iriomote cat and the leopard cat, *Felis bengalensis*, based on ribosomal DNA.** *Jpn J Genet* 1994, **69:**397–406.
42. Todd NB: **Karyotypic fissioning and canid phylogeny.** *J Theor Biol* 1970, **26:**445–480.

43. Wurster-Hill DH, Centerwall WR: **The interrelationships of chromosome banding patterns in canids, mustelids, hyena, and felids.** *Cytogenet Cell Genet* 1982, **34:**178–192.
44. Hunt RM, Jr., Tedford RH: **Phylogenetic relationships within the aeluroid Carnivora and implications of their temporal and geographic distribution.** In *Mammalian phylogeny: placentals.* Edited by Szalay FS, Novacek MJ, McKenna MC. New York: Springer-Verlag; 1993: 53–73
45. Kratochvíl J: **Os penis der Gattung *Panthera* und das System der Felidae (Mammalia).** *Zool Listy* 1976, **25:**289–302.
46. Taylor ME, Matheson J: **A craniometric comparison of the African and Asian mongooses in the genus *Herpestes* (Carnivora: Hespestidae).** *Mammalia* 1999, **63:**449–464.
47. Wesley-Hunt GD, Flynn JJ: **Phylogeny of the Carnivora: basal relationships among the carnivoramorphans, and assessment of the position of "Miacoidea" relative to crown-clade Carnivora.** *J Syst Palaeontol* 2005, **3:**1–28.
48. Dragoo JW, Honeycutt RL, Schmidly DJ: **Taxonomic status of white-backed hog-nosed skunks, genus Conepatus (Carnivora : Mephitidae).** *J Mammal* 2003, **84:**159–176.
49. Brunner S: **Fur seals and sea lions (Otariidae): identification of species and taxonomic review.** *Systematics and Biodiversity* 2003, **1:**339–439.
50. Veron G: **The phylogenetic position of *Cryptoprocta ferox* (Carnivora). Cladistic analysis of the morphological characters of modern and fossil Aeluroidea Carnivora.** *Mammalia* 1995, **59:**551–582.

51. Huo S, Yang J, Xiang Z, Ma S: **Cladistic analysis of the family Viverridae (Carnivora) from China.** *Zool Res* 2003, **24:**413–420.
52. Gaubert P, Wozencraft WC, Cordeiro-Estrela P, Veron G: **Mosaics of convergences and noise in morphological phylogenies: what's in a viverrid-like carnivoran?** *Syst Biol* 2005, **54:**865–894.
53. Flynn JJ, Nedbal MA: **Phylogeny of the Carnivora (Mammalia): congruence vs incompatibility among multiple data sets.** *Mol Phylogenet Evol* 1998, **9:**414–426.
54. Zrzavy J, Ricankova V: **Phylogeny of recent Canidae (Mammalia, Carnivora): relative reliability and utility of morphological and molecular datasets.** *Zool Scr* 2004, **33:**311–333.
55. Wang X, Tedford RH, Van Valkenburgh B, Wayne RK: **Ancestry: evolutionary history, molecular systematics, and evolutionary ecology of Canidae.** In *The Biology and Conservation of Wild Canids.* Edited by Macdonald DW, Sillero-Zubiri C. Oxford: Oxford University Press; 2004: 39–54
56. Koepfli KP, Gompper ME, Eizirik E, Ho CC, Linden L, Maldonado JE, Wayne RK: **Phylogeny of the Procyonidae (Mammalia: Carnivora): molecules, morphology and the Great American Interchange.** *Mol Phylogenet Evol* 2007, **43:**1076–1095.
57. Wyss AR: **On "retrogression" in the evolution of the Phocinae and phylogenetic affinities of the monk seals.** *Am Mus Novit* 1988, **2924:**1–38.
58. Sarich VM: **Pinniped phylogeny.** *Syst Zool* 1969, **18:**416–422.
59. Sarich VM: **Pinniped origins and the rate of evolution of carnivore albumins.** *Syst Zool* 1969, **18:**286–295.

60. Morejohn GV: **A phylogeny of otariid seals based on morphology of the baculum.** *Rapp P-v Réun Cons int Explor Mer* 1975, **169:**49–56.
61. Ling JK: **Pelage characteristics and systematic relationships in the Pinnipedia.** *Mammalia* 1978, **42:**305–313.
62. Kim KC, Repenning CA, Morejohn GV: **Specific antiquity of the sucking lice and evolution of otariid seals.** *Rapp P-v Réun Cons int Explor Mer* 1975, **169:**544–549.
63. Hendey QB: **The evolution and dispersal of the Monachinae (Mammalia: Pinnipedia).** *Ann S Afr Mus* 1972, **59:**99–113.
64. Burns JJ, Fay FH: **Comparative morphology of the skull of the Ribbon seal, *Histriophoca fasciata*, with remarks on the systematics of Phocidae.** *J Zool* 1970, **161:**363–394.
65. Berta A, Wyss AR: **Pinniped phylogeny.** In *Contributions in marine mammal paleontology honoring Frank C Whitmore, Jr. Volume* 29. Edited by Berta A, Deméré TA. San Diego: Proceedings of the San Diego Society of Natural History; 1994: 33–56
66. Tedford RH: **Relationships of pinnipeds to other carnivores (Mammalia).** *Syst Zool* 1976, **25:**363–374.
67. Thenius E: **Zur systematischen und phylogenetischen Stellung des Bambusbären: *Ailuropoda melanoleuca* David (Carnivora, Mammalia).** *Z Säugetierkd* 1979, **44:**286–305.
68. Seal US, Phillips NI, Erickson AW: **Carnivora systematics: immunological relationships of bear serum albumin.** *Comp Biochem Physiol* 1970, **32:**33–48.

69. Nash WG, O'Brien SJ: **A comparative chromosome banding analysis of the Ursidae and their relationship to other carnivores.** *Cytogenet Cell Genet* 1987, **45:**206–212.
70. Ginsburg L: **Sur la position systématique du petit panda, *Ailurus fulgens* (Carnivora, Mammalia).** *Géobios Mém Spéc* 1982, **6:**247–258.
71. Bugge J: **The cephalic arterial system in carnivores, with special reference to the systematic classification.** *Acta Anat* 1978, **101:**45–61.
72. Werdelin L: **Carnivoran ecomorphology: a phylogenetic perspective.** In *Carnivore behavior, ecology, and evolution. Volume* 2. Edited by Gittleman JL. Ithaca, New York: Cornell University Press; 1996: 582–624
73. Wozencraft WC: **The phylogeny of the Recent Carnivora.** In *Carnivore behavior, ecology, and evolution. Volume* 1. Edited by Gittleman JL. Ithaca, New York: Cornell University Press; 1989: 495–535
74. Berta A, Deméré T: ***Callorhinus gilmorei* n. sp. (Carnivora: Otariidae) from the San Diego Formation (Blancan) and its implications for otariid phylogeny.** *Trans S Diego Soc Nat Hist* 1986, **21:**111–126.
75. Bryant HN, Russell AP, Fitch WD: **Phylogenetic relationships within the extant Mustelidae (Carnivora): appraisal of the cladistic status of the Simpsonian subfamilies.** *Zool J Linn Soc* 1993, **108:**301–334.
76. Collier GE, O'Brien SJ: **A molecular phylogeny of the Felidae: immunological distance.** *Evolution* 1985, **39:**473–487.
77. Wyss AR, Flynn JJ: **A phylogenetic analysis and definition of the Carnivora.** In *Mammalian phylogeny: placentals.* Edited by Szalay FS, Novacek MJ, McKenna MC. New York: Springer-Verlag; 1993: 32–52

78. Couturier J, Dutrillaux B: **Evolution chromosomique chez les Carnivores.** *Mammalia* 1985, **50A:**124–162.
79. Flynn JJ, Neff NA, Tedford RH: **Phylogeny of the Carnivora.** In *The phylogeny and classification of the Tetrapods. Volume* 2 — Mammals. Edited by Benton MJ. Oxford: Clarendon Press; 1988: 73–116
80. Fredga K: **Comparative chromosome studies in mongooses (Carnivora, Viverridae).** *Hereditas* 1972, **71:**1–74.
81. Veron G, Catzeflis FM: **Phylogenetic relationships of the endemic Malagasy carnivore *Cryptoprocta ferox* (Aeluroideae): DNA/DNA hybridization experiments.** *J Mamm Evol* 1993, **1:**169–185.
82. Sarich VM: **Transferrin.** *Trans Zool Soc Lond* 1976, **33:**165–171.
83. Wozencraft WC: **A phylogenetic reappraisal of the Viverridae and its relationship to other Carnivora.** *Ph.D. dissertation.* University of Kansas, Lawrence, 1984.
84. Barnes LG, Domning DP, Ray CE: **Status of studies on fossil marine mammals.** *Mar Mamm Sci* 1985, **1:**15–53.
85. de Muizon C: **Phocid phylogeny and dispersal.** *Ann S Afr Mus* 1982, **89:**175–213.
86. Goldman D, Giri PR, O'Brien SJ: **Molecular genetic-distance estimates among the Ursidae as indicated by one- and two-dimensional protein electrophoresis.** *Evolution* 1989, **43:**282–295.
87. Pecon Slattery J, Johnson WE, Goldman D, O'Brien SJ: **Phylogenetic reconstruction of South American felids defined by protein electrophoresis.** *J Mol Evol* 1994, **39:**296–305.

88. Pecon Slattery J, O'Brien SJ: **Molecular phylogeny of the red panda (*Ailurus fulgens*).** *J Hered* 1995, **86:**413–422.

89. Bogdanov LV, Pastukhov VD: **[New data on the taxonomic position of the Baikal seal *Phoca* (*Pusa*) *sibirica* Gmel.].** In *[Morpho-physiological and ecological studies of the Baikal seal]*. Edited by Pastukhov VD. Novosibirsk: Nauka; 1982: 7–12

90. Vrana PB, Milinkovitch MC, Powell JR, Wheeler WC: **Higher level relationships of the arctoid Carnivora based on sequence data and "total evidence".** *Mol Phylogenet Evol* 1994, **3:**47–58.

91. Hofmann O, Braunitzer G: **The primary structure of the hemoglobin of spectacled bear (*Tremarctos ornatus*, Carnivora).** *Biol Chem Hoppe-Seyler* 1987, **368:**949–954.

92. Nojima T: **A morphological consideration of the relationships of Pinnipedia to other carnivorans based on the bony tentorium and bony falx.** *Mar Mamm Sci* 1990, **6:**54–74.

93. Arnason U, Widegren B: **Pinniped phylogeny enlightened by molecular hybridizations using highly repetitive DNA.** *Mol Biol Evol* 1986, **3:**356–365.

94. Hunt RM, Jr.: **The auditory bulla in Carnivora: an anatomical basis for reappraisal of carnivore evolution.** *J Morphol* 1974, **143:**21–76.

95. Arnason U: **The relationship between the four principal pinniped karyotypes.** *Hereditas* 1977, **87:**227–242.

96. Werdelin L: **The evolution of lynxes.** *Ann Zool Fenn* 1981, **18:**37–71.

97. Taylor PJ, Campbell GK, Meester JAJ, Van Dyk D: **A study of allozyme evolution in African mongooses (Viverridae: Herpestinae).** *Z Säugetierkd* 1991, **56:**135–145.
98. Tumlison R, McDaniel VR: **A description of the baculum of the bobcat (*Felis rufus*), with comments on its development and taxonomic implications.** *Can J Zool* 1984, **62:**1172–1176.
99. Trillmich F, Majluf P: **First observations on colony structure, behavior, and vocal repertoire of the South American fur seal (*Arctocephalus australis* Zimmermann, 1783) in Peru.** *Z Säugetierkd* 1981, **46:**310–322.
100. Kratochvíl J: **Karyotyp und System der Familie Felidae (Carnivora, Mammalia).** *Folia Zool* 1982, **31:**289–304.
101. Schmidt-Kittler V: **Zur Stammesgeschichte der marderverwandten Raubtiergruppen (Musteloidea, Carnivora).** *Eclogae Geol Helv* 1981, **74:**753–801.
102. Petter G: **Rapports phylétiques des Viverridés (Carnivores Fissipèdes). Les formes de Madagascar.** *Mammalia* 1974, **38:**605–636.
103. Petter G: **Origine, phylogenie et systématique des blaireaux.** *Mammalia* 1971, **35:**567–597.
104. Anderson E: **Quaternary evolution of the Genus *Martes* (Carnivora, Mustelidae).** *Acta Zool Fenn* 1970, **130:**1–132.
105. Hartl GB, Willing R, Grillitsch M, Klansek E: **Biochemical variation in Mustelidae: are carnivores genetically less variable than other mammals.** *Zool Anz* 1988, **221:**81–90.
106. Braunitzer G, Hofmann O: **Les hémoglobins des pandas.** *C R Séances Soc Biol Fil* 1987, **181:**116–121.

107. Wayne RK, O'Brien SJ: **Allozyme divergence within the Canidae.** *Syst Zool* 1987, **36:**339–355.

108. McKenna MC: **The alpha crystallin A chain of the eye lens and mammalian phylogeny.** *Ann Zool Fenn* 1991, **28:**349–360.

109. de Jong WW: **Protein sequence evidence for monophyly of the carnivore families Procyonidae and Mustelidae.** *Mol Biol Evol* 1986, **3:**276–281.

110. Obara Y: **Karyosystematics of the mustelid carnivores of Japan.** *Honyurui Kagaku* 1991, **30:**197–220.

111. Garland T, Jr, Dickerman AW, Janis CM, Jones JA: **Phylogenetic analysis of covariance by computer simulation.** *Syst Biol* 1993, **42:**265–292.

112. Tedford RH, Taylor BE, Wang X: **Phylogeny of the Caninae (Carnivora: Canidae): the living taxa.** *Am Mus Novit* 1995, **3146:**1–37.

113. Tagle DA, Miyamoto MM, Goodman M, Hofmann O, Braunitzer G, Göltenboth R, Jalanka H: **Hemoglobin of pandas: phylogenetic relationships of carnivores as ascertained with protein sequence data.** *Naturwissenschaften* 1986, **73:**512–514.

114. Werdelin L, Solounias N: **The Hyaenidae: taxonomy, systematics and evolution.** *Fossils Strata* 1991, **30:**1–104.

115. Chiarelli AB: **The chromosomes of the Canidae.** In *The wild canids: their systematics, behavioral ecology and evolution.* Edited by Fox MW. New York: Van Nostrand Reinhold Company; 1975: 40–53

116. Werdelin L: **Morphological patterns in the skulls of cats.** *Biol J Linn Soc* 1983, **19:**375–391.

117. Radinsky L: **Viverrid neuroanatomy: phylogenetic and behavioral implications.** *J Mammal* 1975, **56:**130–150.

118. Peters G: **Acoustic communication in the genus *Lynx* (Mammalia: Felidae) — comparative survey and phylogenetic interpretation.** *Bonner zoologische Beiträge* 1987, **38:**315–330.

119. Ahmed A, Jahan M, Braunitzer G: **Carnivora: the primary structure of hemoglobin from adult coati (*Nasua nasua rufa*, Procyonidae).** *J Protein Chem* 1990, **9:**23–29.

120. Langguth A: **Ecology and evolution in the South American canids.** In *The wild canids: their systematics, behavioral ecology and evolution.* Edited by Fox MW. New York: Van Nostrand Reinhold Company; 1975: 192–206

121. Beentjes MP: **Comparative terrestrial locomotion of the Hooker's sea lion (*Phocarctos hookeri*) and the New Zealand fur seal (*Arctocephalus forsteri*): evolutionary and ecological implications.** *Zool J Linn Soc* 1990, **98:**307–325.

122. Holmes T, Jr.: **Sexual dimorphism in North American weasels with a phylogeny of the Mustelidae.** *Ph.D. dissertation.* University of Kansas, Lawrence, 1988.

123. van Zyll de Jong CG: **A phylogenetic study of the Lutrinae (Carnivora; Mustelidae) using morphological data.** *Can J Zool* 1987, **65:**2536–2544.

124. Schreiber A, Puschmann W, Tichy H: **On the taxonomy of Amur cat (*Felis bengalensis euptilura*).** *Z Säugetierkd* 1993, **58:**13–17.

125. Stirling I, Warneke RM: **Implications of a comparison of the airborne vocalizations and some aspects of the behaviour of the two Australian fur seals, *Arctocephalus* spp., on the evolution and present taxonomy of the genus.** *Aust J Zool* 1971, **19:**227–241.

126. Sarich VM: **Pinniped systematics: immunological comparisons of their albumins and transferrins.** *Am Zool* 1975, **15:**826.

127. Taylor ME: **Foot structure and phylogeny in the Viverridae (Carnivora).** *J Zool* 1988, **216:**131–139.

128. Herrington SJ: **Systematics of the Felidae: a quantitative analysis.** *M.Sc. thesis*. University of Oklahoma, Norman, 1983.

129. Herrington SJ: **Phylogenetic relationships of the wild cats of the world.** *Ph.D. dissertation*. University of Kansas, Lawrence, 1986.

130. Anbinder EM: **Chromosomal sets of the Greenland, Caspian and Baikal seals and some problems of the evolution of true seals (Phocidae).** *Tsitologiya* 1971, **13:**341–347.

131. Arnason U, Ledje C: **The use of highly repetitive DNA for resolving cetacean and pinniped phylogenies.** In *Mammalian phylogeny: placentals. Volume* 3. Edited by Szalay FS, Novacek MJ, McKenna MC. New York: Springer-Verlag; 1993: 74–80

132. Czelusniak J, Goodman M, Moncrief ND, Kehoe SM: **Maximum parsimony approach to construction of evolutionary trees from aligned homologous sequences.** *Methods Enzymol* 1990, **183:**601–615.

133. Hashimoto T, Otaka E, Adachi J, Mizuta K, Hasegawa M: **The giant panda is closer to a bear, judged by α- and β-hemoglobin sequences.** *J Mol Evol* 1993, **36:**282–289.

134. Hemmer H: **The evolutionary systematics of the living Felidae: present status and current problems.** *Carnivore* 1978, **1:**71–79.

135. Hunt RM, Jr., Barnes LG: **Basicranial evidence for ursid affinity of the oldest pinnipeds.** In *Contributions in marine mammal paleontology honoring*

Frank C Whitmore, Jr. Volume 29. Edited by Berta A, Deméré TA. San Diego: Proceedings of the San Diego Society of Natural History; 1994: 57–67

136. Lushnikova T, Omelyanchuk LV, Graphodatsky AS, Radjabli SI, Ternovskaya YG, Ternovsky DV: **Phylogenetic relationships of closely related species (Mustelidae). Interspecific variability of blot-hybridization patterns of BamHI repeats.** *Genetika* 1989, **25:**1089–1094.
137. Modi WS, O'Brien SJ: **Quantitative cladistic analysis of chromosome banding among species in three orders of mammals: hominoid primates, felids and arvicolid rodents.** In *Chromosome structure and function: impact of new concepts.* Edited by Gustafson JP, Appels R. New York: Plenum Press; 1988: 215–242
138. Bininda-Emonds ORP, Russell AP: **A morphological perspective on the phylogenetic relationships of the extant phocid seals (Mammalia: Carnivora: Phocidae).** *Bonn Zool Monogr* 1996, **41:**1–256.
139. Bininda-Emonds ORP, Decker-Flum DM, Gittleman JL: **The utility of chemical signals as phylogenetic characters: an example from the Felidae.** *Biol J Linn Soc* 2001, **72:**1–15.
140. O'Brien SJ, Nash WG, Wildt DE, Bush ME, Benveniste RE: **A molecular solution to the riddle of the giant panda's phylogeny.** *Nature* 1985, **317:**140–144.
141. Gaubert P, Veron G, Tranier M: **Genets and "genet-like" taxa (Carnivora, Viverrinae): phylogenetic analysis, systematics and biogeographic implications.** *Zool J Linn Soc* 2002, **134:**317–334.
