# Supplementary material for: Updating the evolutionary history of Carnivora (Mammalia): a new species-level supertree complete with divergence time estimates
Source: BMC Biol. 2012 Feb 27;10:12. doi: 10.1186/1741-7007-10-12 (PMC3307490; doi:10.1186/1741-7007-10-12)
Supplement: Additional file 6 — Optimal models of evolution for the 74 gene data sets for the relDate analyses. Analyses used autoMT.pl in conjunction with PhyML ([125,126]; nonclock analyses) and PAUP* (clock analyses). An asterisk behind the likelihood ratio test P value indicates that the data set did not evolve according to a strict molecular clock at the 0.05 level (corrected for multiple comparisons). [file 1741-7007-10-12-S6.PDF]

Additional file 6

Optimal models of evolution for the 74 gene data sets for the relDate analyses as determined using autoMT.pl in conjunction with PhyML (nonclock analyses) and PAUP* (clock analyses). An asterisk behind the likelihood ratio test (LRT) *P* value indicates that the data set did not evolve according to a strict molecular clock at the 0.05 level (corrected for multiple comparisons).

**Data set**	**Taxon number**	**Aligned length**	**Optimal model**	**-LnL (nonclock)**	**-LnL (clock)**	**χ^2 value**	**LRT *P* value**
ALAS	43	514	HKY+G	1780.0065	1808.51239	57.012	4.94×10^{-2} *
APOB (exon 26)	58	963	GTR+G	5184.9424	5227.90924	85.934	6.20×10^{-3} *
APOB (exon 29, part 1)	36	702	HKY+G	3154.1079	3196.26902	84.322	3.68×10^{-6}
APOB (exon 29, part 2)	60	633	TVM+G	3960.6335	4037.34162	153.420	1.48×10^{-10}
APP	49	679	TVM+G	2723.1899	2761.5891	76.798	3.93×10^{-3} *
ATPA7	54	675	K81uf+G	3387.8828	3443.95148	112.140	2.65×10^{-6}
BNDF	62	753	TrN+G	11177.3242	21374.47846	20394.000	0.00
BRCA1 (part 1)	40	705	TVM+I	3663.2146	3683.2823	40.135	3.76×10^{-1} *

Data set	Taxon number	Aligned length	Optimal model	-LnL (nonclock)	-LnL (clock)	χ^2 value	LRT *P* value
BRCA1 (part 2)	41	729	TVM+G	4322.2432	4360.07145	75.657	3.91×10^{-4}
CALB	38	822	TVM+G	1979.6913	1998.8913	38.400	3.61×10^{-1} *
CHST12	37	705	TVM+G	2241.5071	2255.62326	28.232	7.84×10^{-1} *
CLU	46	1422	TVMef+G	6008.2798	6063.99597	111.430	9.10×10^{-8}
CMA1	46	292	HKY+G	1793.3361	1819.62748	52.583	1.76×10^{-1} *
CXCR7	37	735	TVM+G	2353.3604	2384.65312	62.585	2.83×10^{-3} *
CYP1A1	25	276	K80+G	939.3657	953.56332	28.395	2.01×10^{-1} *
DGKG	41	751	GTR+G	2636.8743	2655.30938	36.870	5.67×10^{-1} *
EPM2B	28	389	TrN+G	1285.6576	1324.89466	78.474	3.60×10^{-7}
FES	42	429	TVM+G	2006.1582	2030.66385	49.011	1.55×10^{-1} *
GATA3	42	452	TVMef+G	1489.8887	1543.26615	106.750	5.48×10^{-8}
GHR	142	933	TIM+G	4946.272	5046.70709	200.870	5.78×10^{-4}

**Data set**	**Taxon number**	**Aligned length**	**Optimal model**	**-LnL (nonclock)**	**-LnL (clock)**	**χ^2 value**	**LRT *P* value**
GNAZ	43	622	TIM+I+G	1718.4282	1740.52317	44.190	3.38×10^{-1} *
GNB1	41	698	GTR+G	3171.9788	3213.55049	83.143	4.94×10^{-5}
HK1	42	378	HKY+G	1690.6991	1726.91972	72.441	1.27×10^{-3}
MT-ATP6	67	681	GTR+I+G	14624.165	14678.27302	108.220	6.16×10^{-4}
MT-ATP8	68	207	GTR+I+G	4885.4019	4925.81359	80.823	1.04×10^{-1} *
MT-CO1	73	1551	GTR+I+G	30857.6289	30923.87907	132.500	1.33×10^{-5}
MT-CO2	88	684	GTR+I+G	14811.5879	14880.31149	137.450	3.57×10^{-4}
MT-CO3	66	804	TIM+I+G	14542.5293	14607.55492	130.050	2.10×10^{-6}
MT-CYB	202	1144	GTR+I+G	50093.0664	50454.79308	723.450	1.62×10^{-58}
MT-ND1	69	957	GTR+I+G	18435.9316	18503.23585	134.610	1.90×10^{-6}
MT-ND2	131	1050	GTR+I+G	41332.7227	41556.98939	448.530	7.11×10^{-36}
MT-ND3	67	357	GTR+I+G	7554.6689	7604.05582	98.774	4.39×10^{-3} *

**Data set**	**Taxon number**	**Aligned length**	**Optimal model**	**-LnL (nonclock)**	**-LnL (clock)**	**χ^2 value**	**LRT *P* value**
MT-ND4	78	1378	GTR+I+G	33395.2109	33618.95023	447.480	0.00
MT-ND4L	67	297	TrN+I+G	6189.3076	6238.45134	98.287	4.83×10^{-3} *
MT-ND5	74	1836	GTR+I+G	43243.0508	43345.51135	204.920	1.09×10^{-14}
MT-ND6	61	534	GTR+I+G	9657.749	9730.24199	144.990	3.41×10^{-9}
MT-RNR1	100	1030	GTR+I+G	17117.4922	17323.83952	412.690	0.00
MT-RNR2	76	1665	GTR+I+G	23220.5234	23356.62664	272.210	1.87×10^{-24}
MT-TA (tRNA-Ala)	54	70	TrN+G	667.5964	706.87499	78.557	1.01×10^{-2} *
MT-TC (tRNA-Cys)	52	69	TVMef+I+G	596.6256	630.77945	68.308	4.36×10^{-2} *
MT-TD (tRNA-Asp)	47	70	HKY+G	617.2423	646.18275	57.881	9.42×10^{-2} *
MT-TE (tRNA-Glu)	57	71	HKY+G	669.9344	711.24126	82.614	9.39×10^{-3} *
MT-TF (tRNA-Phe)	56	74	TrN+G	757.1855	802.22143	90.072	1.50×10^{-3} *
MT-TG (tRNA-Gly)	53	73	GTR+G	993.1248	1028.05689	69.864	4.08×10^{-2} *

**Data set**	**Taxon number**	**Aligned length**	**Optimal model**	**-LnL (nonclock)**	**-LnL (clock)**	**χ^2 value**	**LRT *P* value**
MT-TH (tRNA-His)	53	73	K81uf+G	715.7496	749.50534	67.511	6.06×10^{-2} *
MT-TI (tRNA-Ile)	53	71	HKY+I+G	379.6047	417.04241	74.875	1.64×10^{-2} *
MT-TK (tRNA-Lys)	55	73	TVM+I+G	958.0111	1001.11433	86.206	2.66×10^{-3} *
MT-TM (tRNA-Met)	53	70	TVMef+I+G	254.4667	279.369	49.805	5.21×10^{-1} *
MT-TN (tRNA-Asn)	48	73	TVM+G	551.0546	579.36688	56.625	1.36×10^{-1} *
MT-TP (tRNA-Pro)	64	71	GTR+G	905.9548	942.18011	72.451	1.71×10^{-1} *
MT-TQ (tRNA-Gln)	54	75	HKY+I+G	816.9633	854.33958	74.753	2.10×10^{-2} *
MT-TR (tRNA-Arg)	52	74	HKY+I+G	706.8306	741.25355	68.846	3.97×10^{-2} *
MT-TT (tRNA-Thr)	63	81	HKY+G	1067.9836	1110.00532	84.043	2.69×10^{-2} *
MT-TV (tRNA-Val)	70	82	GTR+G	1256.2465	1319.54626	126.600	2.10×10^{-5}
MT-TW (tRNA-Trp)	54	72	TrN+I+G	755.6812	794.29091	77.219	1.32×10^{-2} *
MT-TY (tRNA-Tyr)	53	71	TIM+G	687.6532	749.20396	123.100	6.75×10^{-8}

**Data set**	**Taxon number**	**Aligned length**	**Optimal model**	**-LnL (nonclock)**	**-LnL (clock)**	**χ^2 value**	**LRT *P* value**
PLP1	43	913	TVM+G	3896.5242	3937.27955	81.511	1.71×10^{-4}
PNOC	67	307	TIM+I+G	2094.3098	2142.052	95.484	8.22×10^{-3} *
RAG1 (part 1)	62	1095	SYM+I+G	6617.7695	6668.92999	102.320	5.45×10^{-4}
RAG1 (part 2)	38	741	TrN+G	2870.8049	2939.43588	137.260	9.66×10^{-14}
RAG2	62	482	TVM+I+G	2266.2412	2288.72489	44.967	9.26×10^{-1} *
RBP3	123	1293	GTR+I+G	12342.4346	12484.48587	284.100	5.70×10^{-15}
RSA2	44	579	HKY+G	1972.6233	1990.49297	35.739	7.41×10^{-1} *
SIL	43	375	K80+G	1281.876	1310.67061	57.589	4.44×10^{-2} *
SRY	69	714	TVM+G	4738.752	4779.30515	81.106	1.15×10^{-1} *
TBG	33	442	TVM+G	2501.3711	2544.00773	85.273	5.80×10^{-7}
TCP1	42	239	SYM+G	843.4976	861.20817	35.421	6.76×10^{-1} *
TEME20	35	615	TVM+G	2599.8792	2623.40424	47.050	5.36×10^{-2} *

Data set	Taxon number	Aligned length	Optimal model	-LnL (nonclock)	-LnL (clock)	χ^2 value	LRT *P* value
TTR	40	886	GTR+G	3067.9204	3085.75114	35.661	5.78×10^{-1} *
VANGL2	37	546	TrN+I+G	2094.7422	2217.39566	245.310	2.11×10^{-33}
VTN	40	224	K80+G	839.8723	861.74579	43.747	2.41×10^{-1} *
VWF	50	1276	TIM+I+G	6284.2402	6340.21839	111.960	5.03×10^{-7}
ZFX	38	1181	GTR+I+G	2933.6865	2969.74569	72.118	3.30×10^{-4}
ZFY	49	1181	GTR+I+G	4105.7681	4160.02217	108.510	9.06×10^{-7}
